# SynKit: A Graph-Based Python Framework for Rule-Based Reaction Modeling and Analysis

**DOI:** 10.1021/acs.jcim.5c02123

**Published:** 2025-12-03

**Authors:** Tieu-Long Phan, Marcos E. González Laffitte, Klaus Weinbauer, Daniel Merkle, Jakob Lykke Andersen, Rolf Fagerberg, Thomas Gatter, Peter F. Stadler

**Affiliations:** † Bioinformatics Group, Department of Computer Science & Interdisciplinary Center for Bioinformatics & School for Embedded and Composite Artificial Intelligence (SECAI), 9180 Leipzig University, Härtelstraße 16−18, D-04107 Leipzig, Germany; ‡ Department of Mathematics and Computer Science, 6174 University of Southern Denmark, DK-5230 Odense M, Denmark; § Center for Scalable Data Analytics and Artificial Intelligence (ScaDS.AI), 9180 Leipzig University, D-04103 Leipzig, Germany; ∥ Machine Learning Research Unit, TU Wien Informatics, A-1040 Wien, Austria; ⊥ Algorithmic Cheminformatics Group, Faculty of Technology & Center for Biotechnology (CeBiTec), Bielefeld University, Postfach 10 01 31, D-33501 Bielefeld, Germany; # Max Planck Institute for Mathematics in the Sciences, Inselstraße 22, D-04103 Leipzig, Germany; ∇ Department of Theoretical Chemistry, University of Vienna, Währingerstraße 17, A-1090 Vienna, Austria; ○ Facultad de Ciencias, Universidad National de Colombia, Bogotá CO-111321, Colombia; ◆ Center for non-coding RNA in Technology and Health, University of Copenhagen, Ridebanevej 9, DK-1870 Frederiksberg, Denmark; ¶ Santa Fe Institute, 1399 Hyde Park Rd., Santa Fe, New Mexico 87501, United States

## Abstract

Computational modeling of chemical reactions is fundamental to modern synthetic chemistry but is often hindered by a fragmented software ecosystem and the complexity of accurately representing the reaction mechanisms. To address this, we introduce SynKit, an open-source Python library that provides a unified, chemically intuitive framework for reaction informatics. SynKit performs core tasks such as reaction canonicalization and transformation classification, while other functionalities—such as synthetic route construction through rule composition—are supported through integration with external libraries. The newly introduced *Mechanistic Transition Graph* extends the traditional net-change representation of the *Imaginary Transition State* by explicitly modeling the sequence of bond-forming and bond-breaking events, capturing transient intermediates, and providing deeper mechanistic insight. Designed for easy installation and broad compatibility, SynKit integrates smoothly into existing computational workflows for exploring complex *Chemical Reaction Networks*. For more advanced network analyses, it interfaces with specialized tools (e.g., MØD) to support exhaustive mechanism enumeration and kinetics-aware studies. By combining advanced mechanistic modeling with an accessible, modular design, SynKit supports more reproducible and rigorous research in automated synthesis planning.

## Graphical abstract

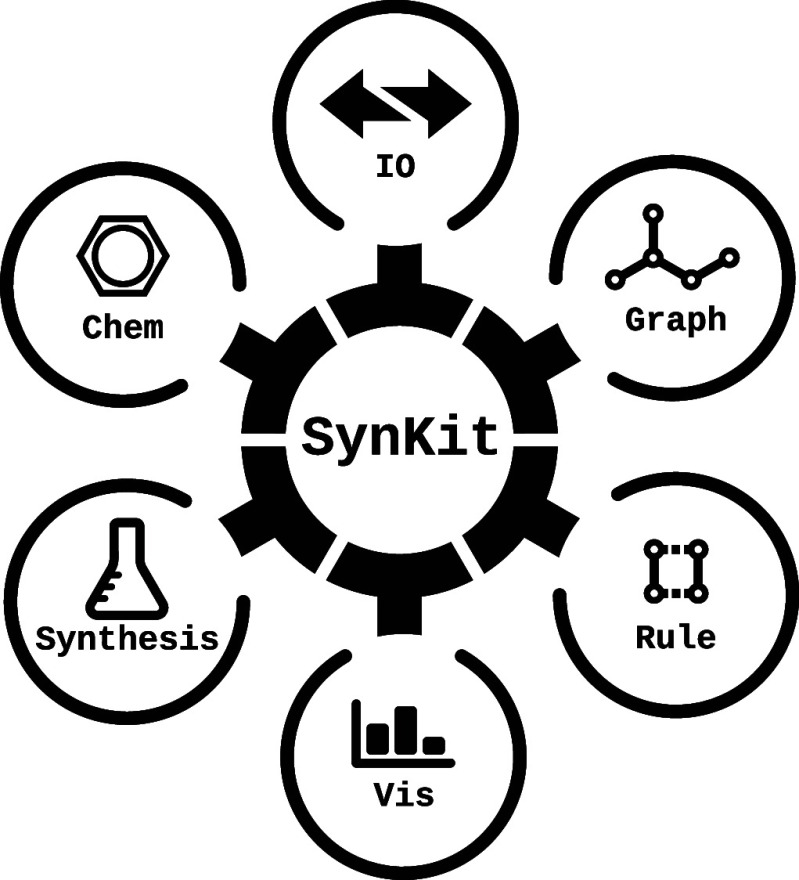

## 1 Introduction

Chemical reaction modeling is central to cheminformatics and computer-aided synthesis planning (CASP).^1,2^ Over the years, a suite of powerful software, such as ChemAxon,^3^ CGRtools,^4^ RDKit,^5^ RDChiral,^6^ Open Babel,^7^ and OpenEye Toolkits,^8^ has been developed to represent, analyze, and predict chemical reactions. These tools enable tasks such as identifying reactive functional groups, assessing reaction similarity, and encoding reaction rules. With continued progress in the field, specialized tools like RDCanon^9^ and SynPlanner^10^ have emerged for tasks such as reaction canonicalization and retrosynthesis planning.

Despite recent advances, reaction informatics remains fragmented, lacking seamless interoperability between tools tailored to narrow chemical tasks. For instance, RDChiral, used in AiZynthFinder,^11^ fails to capture the full topological and electronic complexity of reaction centers^6^ in SMARTS templates. CGRtools employs condensed graphs that limit atom-level mechanistic fidelity for tasks such as resolving arrow-pushing or tracking transient intermediates.^4^ While RDKit, the core chemical engine of REINVENT,^12^ excels at molecular manipulation, its reaction canonicalization is confined to lexicographic SMILES ordering, lacks semantically consistent hashing, and offers no abstraction for multistep reaction pathways.^13^ This fragmentation impedes reproducible and automated synthesis by distracting it from the core chemistry.

To address this fragmentation and the adoption barriers that prevent experimentalists and nonexpert users from harnessing the full potential of reaction informatics, we introduce SynKit, an extensible Python toolkit that unifies reaction informatics. It is designed to bridge disparate computational tools with atom-level mechanistic modeling and is not intended to replace specialized software; in particular, it is not a synthesis planner. More than a simple wrapper, SynKit offers Python reimplementations of core algorithms, built upon open-source software RDKit and NetworkX. It mitigates integration fragility via a unified data schema, an adapter-based I/O layer for format conversion, and a modular plugin system with optional compiled backends for performance. SynKit offers features such as reaction canonicalization, template clustering and classification, and the construction of novel synthetic routes. These capabilities are powered by a rule-based framework built on the *Double Pushout* (DPO) graph transformation formalism.^14^ Beyond workflow support, SynKit extends the *Imaginary Transition State* (ITS)^15^ and *Condensed Graph of Reaction* (CGR)^4^ representations into *Mechanistic Transition Graphs* (MTGs). In more detail, MTGs leverage the rule-composition formalism^16,17^ to encode stepwise mechanisms using per-bond bond-order vectors and atom-state change records, resembling the *overlay graph* framework^18^ but preserving temporal order.

## 2 Software Overview

SynKit is a Python software package for modeling chemical reactions and reaction networks that links to mature open-source libraries. It relies on RDKit^5^ for molecular and reaction processing. For graph algorithms, SynKit defaults to NetworkX,^19^ ensuring ease of installation and broad cross-platform compatibility. An optional MØD backend^17^ offers advanced graph-transformation and rule-composition features at the cost of a more involved, platform-dependent setup and GPL licensing implications.[Fn fn1] SynKit is released under the MIT License and is available on PyPI, Conda, and DockerHub (see Supporting Section A).

The architecture of SynKit is modular, divided into six core subpackages (see Figure S1 and Table 1): IO (parsing and format conversion), Chem (standardization, canonicalization, atom-map comparison), Graph (canonicalization, ITS/MTG construction, isomorphism, clustering, and subgraph search), Rule (DPO and graph-based rule composition), Synthesis (forward/backward prediction, chemical reaction network exploration, pathway analysis), and Vis (molecule and mechanism visualization).

**Table 1:**
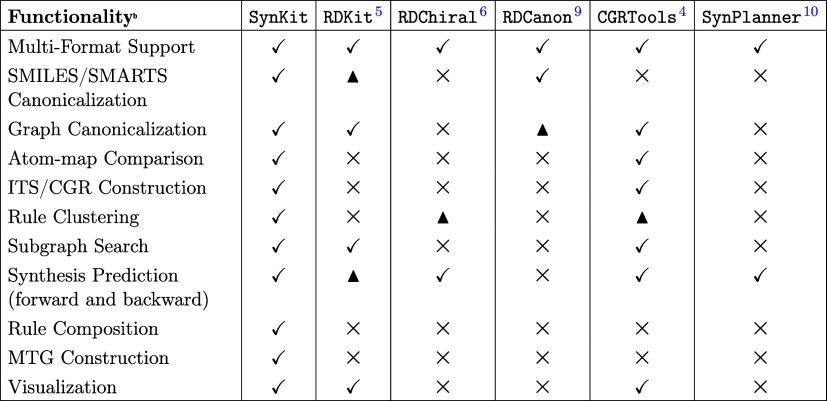
Feature Comparison of SynKit and Other Reaction Modeling Tools^a^

a Legend: 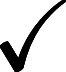 Full, ▲ Partial, and 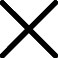 None.

b The listed packages provide broader functionality beyond this work (e.g., RDKit includes conformer generation, pharmacophore and alignment tools, SynPlanner extends CGRtools for retrosynthesis). The table reports only SynKit’s native core features and highlights its bridging role to specialist tools and format conversion. See Supporting Section B for details.

## 3 Software Features and Performance Evaluation

The notation follows our previous work,^20,21^ while full mathematical details are in Supporting Section C. We benchmark SynKit on conversion, canonicalization, template clustering, and subgraph search using 39,732 atom-mapped reactions from USPTO_50k^22^ preprocessed with SynTemp,^20^ while also assessing canonicalization on USPTO_3k.^20,23^ All experiments were run in Python 3.11 on a 12-core Intel i7-8700 (3.20 GHz) under Fedora 37.

### 3.1 IO Module for Chemical Conversions

The computational modeling of chemical reactions uses many cheminformatics toolkits with different representations. RDChiral^6^ uses SMARTS/SMILES templates to encode transformations, whereas graph-based tools model reactions explicitly: CGRtools^4^ builds Condensed Graphs of Reaction to represent bonding changes, and SynTemp^17,20^ employs ITS graphs and GML-encoded rules. The IO subpackage—a bidirectional translator between linear notations (reaction SMILES) and graph formats (ITS graphs, GML)—bridges these individual formalisms (Figure 1A). While the implementation keeps explicit hydrogens at reaction centers for atom-mapping and valency accuracy, it omits stereochemistry. Benchmarks on USPTO_50k show high-throughput conversion: SMILES → GML averages 2.19 ± 2.15 ms/reaction and GML → SMILES averages 3.63 ± 2.69 ms. These conversions are lossless for balanced reactions, but missing reaction-center hydrogens can prevent exact reversion from GML to SMILES. Full results are provided in Supporting Section D.

**Figure 1:**
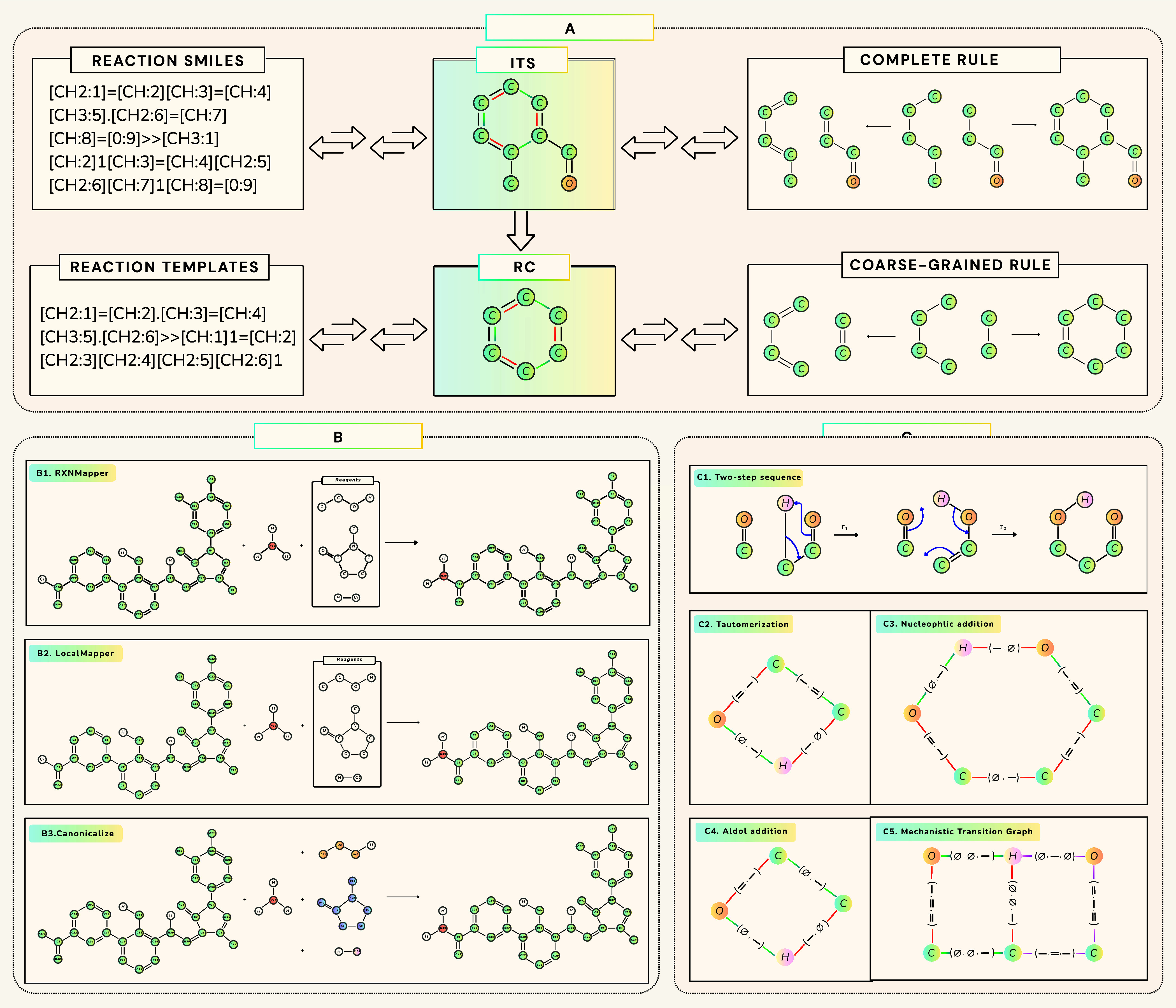
Conversion, canonicalization, and MTG construction in SynKit. (A) Primary formats, including reaction SMILES, ITS graphs (NetworkX), and DPO rules (GML), are converted bidirectionally while preserving atom-mapping and provenance. ITS graphs can be reduced to reaction-center representations. (B) Atom-map canonicalization: (B1) RXNMapper;^24^ (B2) LocalMapper;^23^ (B3) canonical form produced by our exact canonicalizer, to which (B1) and (B2) map. (C) MTG example for a two-step aldol addition reaction: (C1) overall sequence; (C2) step-1 center (tautomerization); (C3) step-2 center (nucleophilic addition); (C4) net reaction center before water elimination; (C5) resulting Mechanistic Transition Graph.

### 3.2 Chem Module

Complementing the format-conversion utilities, the Chem module provides a compact toolkit for fine-grained reaction analysis: reaction standardization, deterministic representation of reaction SMARTS/SMILES, and atom-mapping comparison. Our standardization extends RDKit’s molecular protocols to full-transformational settings and includes tautomer normalization to reduce false negatives in atom-mapping comparisons. We also adapt the AAMValidator interface from SynTemp^20^ to enable robust, reproducible atom-mapping checks built on the Chem utilities.

The GraphCanonicaliser (Section 3.3.1) performs atom-map canonicalization (Figure 1B) by standardizing reactants and refining products based on atom maps. SynKit provides an exact individualization-refinement canonicalizer based on Nauty^25^ and Bliss^26^ (Algorithm S1), while correctness is validated by canonical SMILES comparison and structural checks with AAMValidator, yielding perfect accuracy on our test set (Table S2). For speed, we offer an approximate Weisfeiler–Lehman graph hash^27,28^ (WLGH, Algorithm S3) implemented in NetworkX, with the WLGH_3_ version (three iterations) being fast (avg. 3.98 ± 1.75 ms, median 0.39 ms) but giving noncanonical outputs in 5% of cases. The exact canonicalizer averages 266.08 ± 26374.77 ms (median 0.77 ms) due to occasional hard instances. RDCanon^9^ fails in 6% of cases. Our exact method matches independent graph-isomorphism checks, while WLGH and RDCanon show higher disagreement rates (Table S2). Compared to CGRtools, our individualization–refinement canonicalizer is more accurate for exact matches, while WLGH_3_ performs similarly in approximate comparisons. WLGH is suitable for high-throughput pipelines if occasional rechecks are acceptable in practice.

### 3.3 Graph Module

#### 3.3.1 Canonicalization

The GraphCanonicaliser implements two methods described above: an exact canonicalizer inspired by Nauty^25^ and Bliss,^26^ and a WLGH for approximate computation of a canonical form. Performance mirrors the AAM canonicalization results: on USPTO_3k ITS graphs, WLGH_3_ runs at 0.77 ± 0.22 ms per graph. The exact canonicalizer’s mean is inflated by rare symmetric outliers (mean 173.56 ± 13,020.55 ms) (see Figure S3A), so medians are more informative: exact median 0.46 ms versus WLGH_3_ median 0.11 ms, i.e., about 4× slower by median. Use the exact method when correctness is essential, while using WLGH for high-throughput approximations (Supporting Section E.1).

#### 3.3.2 Imaginary Transition State

The *ITS* graph^15,29^ merges reactants and products into a single supergraph that captures net transformations: bond cleavage, formation, and bond-order change. ITS graphs can be mined to extract reaction templates or translated into DPO graph transformation rules^17,20^ for retrosynthesis and reaction-network analysis. In SynKit, ITS graphs are constructed via a dual interface: a high-level IO API for rapid SMILES/SMARTS → ITS conversion and a low-level Graph.ITSConstruction class for fine-grained control and reaction center curation.

To represent multistep mechanisms, SynKit builds *Mechanistic Transition Graphs* (MTGs) by stitching stepwise reaction centers through graph-merge operations that align overlapping fragments and intermediates and unify transformation rules, yielding an approach analogous to the *Overlay Graph* concept.^18^ Each MTG annotates bonds and atom-state attributes (charge, radical) with per-step vectors of length *n* + 1 for an *n*-step transformation, thereby preserving event order and enabling detailed mechanistic interpretation as well as quantitative comparison of alternative decompositions. The MTG yields a graph encoding that links starting materials, intermediates, and products, enabling pathway analysis and mechanism-aware template extraction. Conceptually, the MTG is a concatenation of ITS for consecutive reaction steps. For a formal point of view, it implicitly encodes all intermediates as well as the ITS graphs connecting two consecutive intermediates. Figure 1C depicts a two-step aldol reaction (C1): the net reaction core (C4) captures only the net transformation and elides the tautomerization, whereas the MTG resolves the mechanistic centers (C2 and C3) and transient (pink) bonds along the reaction pathway to the product (C5). For more details, see Supporting Section E.2.

#### 3.3.3 Graph Matcher

From a theoretical perspective, clustering reaction templates reduces to constrained graph-isomorphism problems because graph representations capture the bond changes of each transformation^20,21,30^ (see Supporting Section C). Exact atom-level comparisons are intractable at scale, so practical pipelines rely on fast heuristics.

We implement GraphCluster, which uses the Weisfeiler–Lehman full graph hash (WL)^27,28^ as a rapid prefilter. WL iteratively aggregates local atomic environments into compact ITS graph fingerprints that group similar reactions and avoid many expensive isomorphism checks (Figure S6A). On USPTO_50k, a single WL iteration reduces clustering time from 268.55 ± 4.14 to 16.33 ± 0.02 min. The WL prefilter does not alter the final clustering because exact isomorphism checks are retained within each WL bucket. A heuristic experiment (Supporting Section E.3.1) shows that WL_4_ attains perfect agreement with VF2: purity,^31^ ARI,^32^ and NMI^33^ all equal 1.0. SynKit yielded 270 fine-grained rules, covering reactions more broadly than RDChiral [Bibr ref6] (1892 canonical templates); however, RDChiral remains superior in stereochemical handling because the current SynKit templating pipeline does not enforce stereochemistry and may conflate distinct stereoisomers. Stereo-aware hashing and an optional strict-equivalence mode based on isomorphism are planned to address this. Moreover, the exact matcher relies on molecular graphs, which cannot represent resonance or tautomerism, so tautomers may appear nonisomorphic. To scale beyond exhaustive all-versus-all comparisons (Algorithm S4), we provide BatchClustering (Algorithm S5), which partitions data, clusters batches, and merges results to build a global classification while keeping memory and compute bounded.

Complementary to clustering, the substructure search in the SubgraphMatch module finds reaction cores and motifs. It performs exact subgraph isomorphism (NetworkX backend) and takes 0.1 ± 0.1 ms per query, which is precise but slow for bulk screening. For high-throughput motif discovery, we provide SING, a reimplementation of Di Natale et al.’s nonhomogeneous search:^34^ node-neighborhood fingerprints produce short candidate lists that a backtracking verifier confirms (Figure S6B). In practice, screening ≈40,000 reactions for 270 query patterns (Figure S7B) completes in about 2.90 ± 0.29 ms per query with SING, cutting total query time by over 50% versus an iterative SubgraphMatch approach. The search produces identical results, and SING currently supports simple undirected graphs (no parallel edges) and noninduced subgraph search, but does not support directed graphs. These combined strategies enable accurate motif detection and scalable clustering for large reaction corpora.

### 3.4 Rule Module

Reaction rules/templates may be written as SMILES/SMARTS or as molecular graphs (NetworkX, GML) (Figure 2). Graph forms are especially suitable for computational synthesis planning (Section 3.5). Following the SynTemp approach,^20^ rules exist at two granularities. “Coarse-grained” rules record only core bond rearrangements for maximal generality (Figure 2B), while “refined” rules include the local atomic environment to a chosen bond radius to capture steric and electronic influences (Figure 2D). Coarse centers can be exported as DPO rules in GML (Figure 2C), enabling efficient template matching in pipelines and indexing. A heuristic experiment in Supporting Section F shows that expansion at *r* = 1 matches the retrieval performance of *r* = 2 at substantially lower cost, so *r* = 1 is chosen as the default.

**Figure 2:**
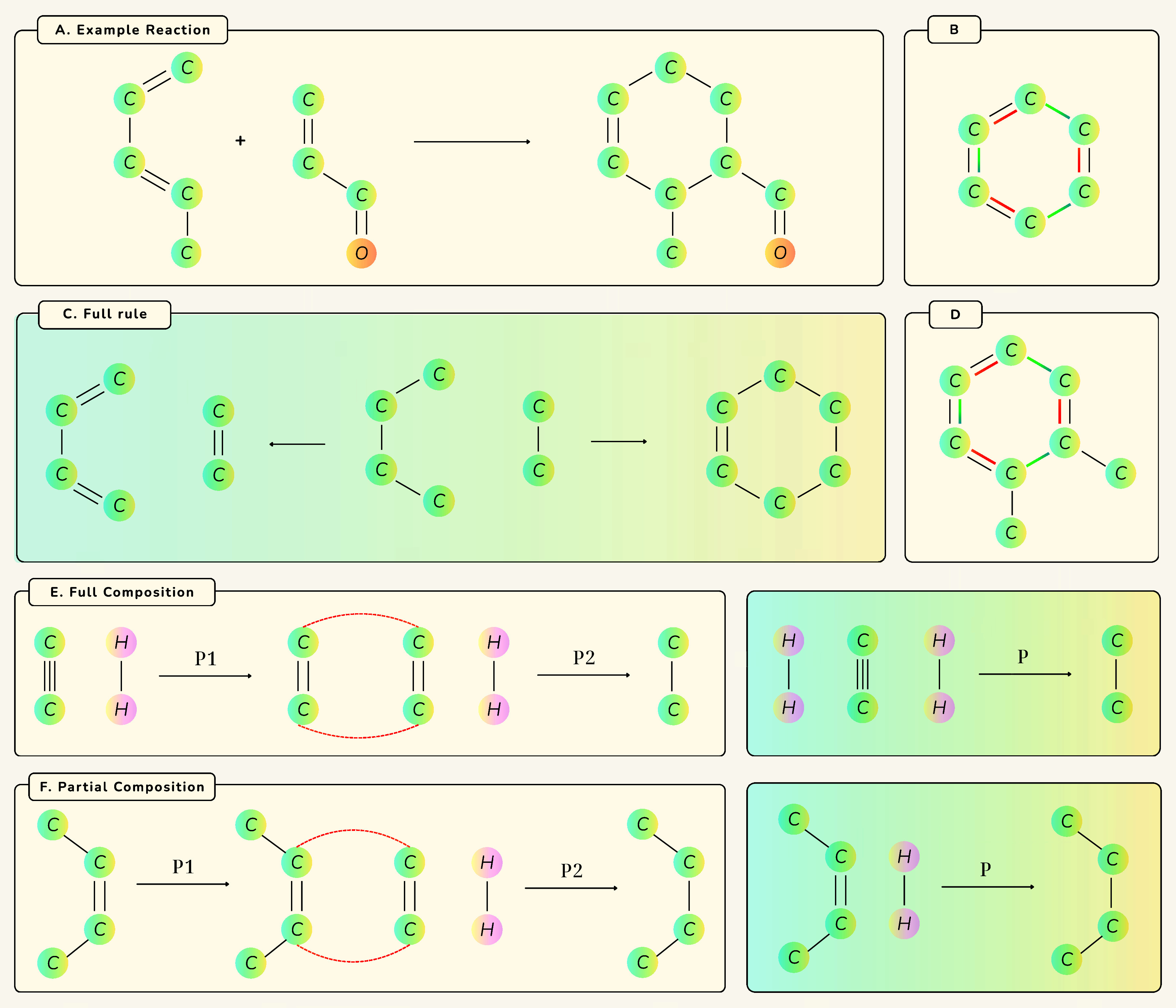
Illustration of reaction-rule representations. (A) Diels–Alder example. (B) Coarse-grained rule in GML. (C) Reaction center template. (D) Reaction center extended by a radius of 1. (E) Rule composition: sequential hydrogenations (alkyne → alkene → alkane). (F) Partial composition: hydrogenation of but-2-ene without explicit H_2_.

SynKit composes sequential templates into a single *composite rule* via the RuleCompose class,^16^ collapsing ordered steps into a net transformation that preserves atom mappings and isotope labels across intermediates and ensures mass- and isotope-level bookkeeping. Composite rules export to NetworkX or GML, enabling efficient template matching, compact indexing in retrosynthetic pipelines, integration with graph-based planners, downstream scoring, and experimental scheduling (see Figure 2E).

SynKit also integrates the partial-composition algorithm from the MØD package^16,17^ to handle transformations that require an external reagent (Section 3.5). For example, since a hydrogenation rule (*p*_2_) is inapplicable to but-2-ene without its H_2_ coreactant, partial composition constructs a composite rule (*p*_3_) for the direct conversion to butane, implicitly sourcing the required hydrogen from the reaction conditions (see Figure 2F).

### 3.5 Synthesis Module

#### 3.5.1 Reactor

Chemical reactions can be modeled as graph transformations using the DPO formalism.^35^ A reaction is defined by a rule span $\left(L\overset{l}{\leftarrow }K\overset{r}{\to }R\right)$ , often from a reaction template. Given a substrate graph *G* and a morphism *m*: *L* → *G*, a series of two Pushout constructions generates the product graph *H*. This reversible transformation aids both forward and backward reaction prediction (Figure 3A).

**Figure 3:**
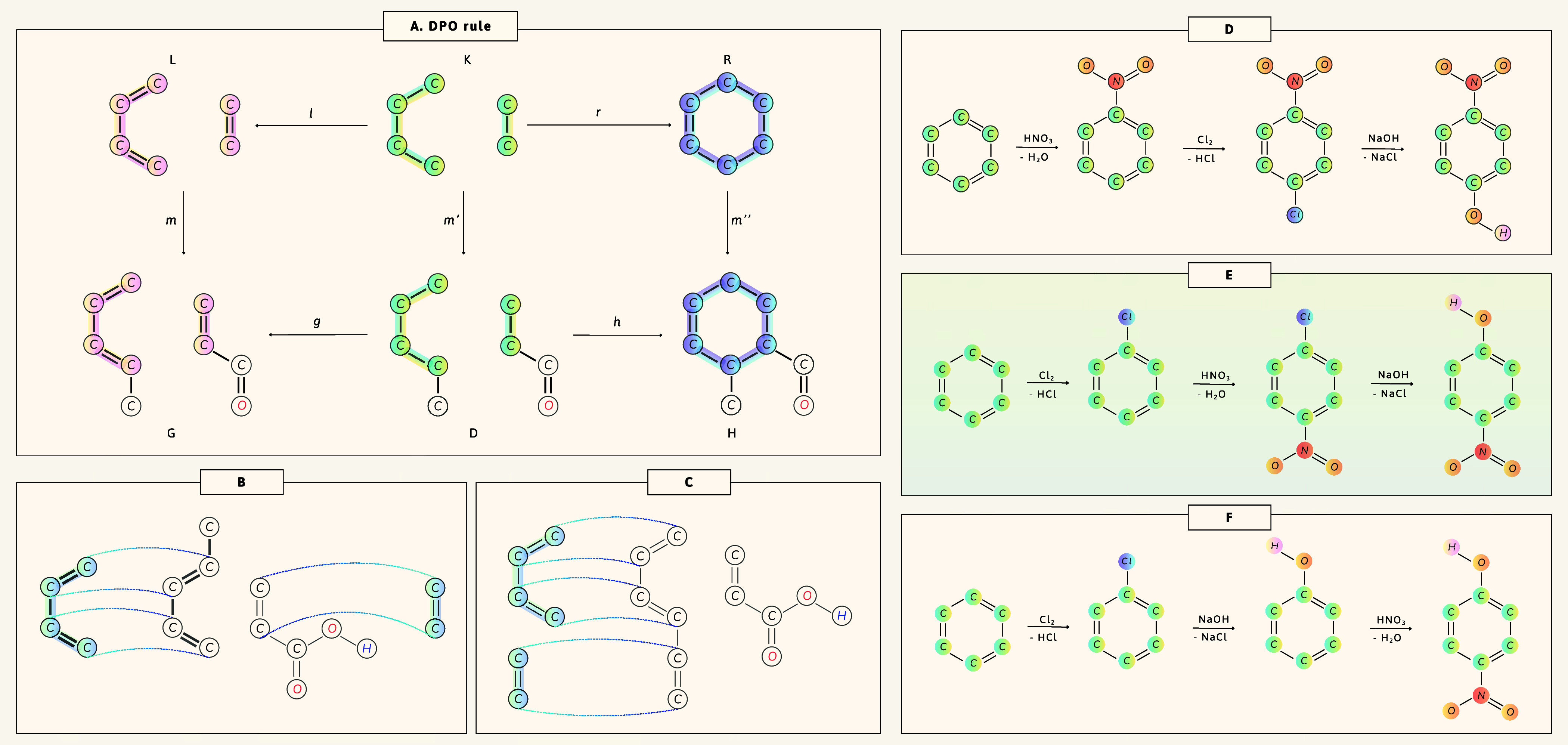
Graph transformations and synthetic routes to *p*-nitrophenol. (A–C) DPO rewriting rule $L\overset{l}{\leftarrow }K\overset{r}{\to }R$ applied to graph *G* yielding product *H*: (A) schematic; (B) component-covering (intramolecular); (C) unconstrained (intermolecular). (D–F) Route examples (no feasibility scoring): (D) chlorination gives *meta* (infeasible); (E) viable—correct ordering yields *para*; (F) nitration of phenol is unselective and oxidizing (infeasible).

A key step is locating the reactive substructure in a reactant molecule *G* (formally a graph morphism *m*: *L* → *G*), performed by the SubgraphSearchEngine. Our *component-covering matching* enforces two constraints: every reactant pattern in the template *L* must be found in the molecular ensemble *G*, and each molecular species in the mixture must contribute at least one matched substructure. This prevents the erroneous application of intermolecular templates to sites within the same molecule and prunes many spurious or chemically infeasible pathways (Figure 3B). For explicit intramolecular modeling (e.g., cyclizations), the constraint may be relaxed to allow unconstrained matching (Figure 3C).

While MØD enables flexible graph transformations, it lacks high-level synthesis planning and poses a steep learning curve for nonexperts. We introduce the Reactor submodule—a Python wrapper that exposes approachable computation backends. SynKit provides two options. SynReactor is a NetworkX-based, DPO-like engine with comprehensive atom-mapping output, native handling of implicit hydrogens, minimal dependencies, and simple installation. MODReactor (*mod*) wraps MØD’s core DPO routines and is computationally efficient for prediction tasks. Benchmarking on the USPTO_50k data set (Table 2, Figure S10) shows both backends preserve transformational fidelity, with MODReactor being faster overall (*p* < 0.05, *t*-test), while SynReactor becomes notably faster when implicit-hydrogen handling is required. While SynKit is not a turnkey retrosynthesis planner since full planning requires curated reaction databases, search-and-scoring strategies, and feasibility models, it supplies modular primitives to assemble custom planning pipelines and link external databases.

**Table 2:**
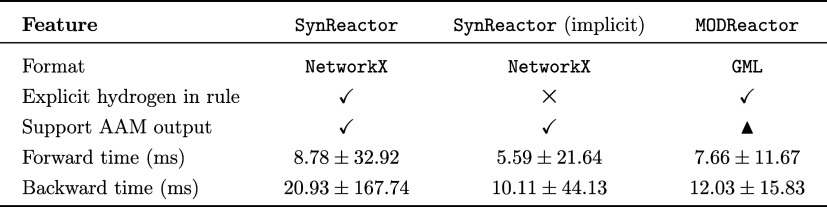
Comparison of SynReactor and MODReactor Backends for Reaction Prediction; Symbols: 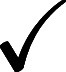 Full, ▲ Partial, and 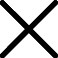 None

In high-throughput screening (e.g., ∼4 × 10^4^ reactants vs 270 templates), the SING prefilter speeds the pure-Python SynReactor up by around 1.7×, while C++ MODReactor remains 2.5× faster. To curb combinatorial explosion, SynReactor uses two safeguards: embed_threshold (hard cap on embeddings, default of 5000) and embed_pre_filter (heuristic discarding templates whose estimated candidate count exceeds the cap). These keep false negatives below 0.05% and resource use tractable.

#### 3.5.2 Chemical Reaction Networks

To simulate the evolution of complex chemical systems, the Synthesis subpackage features a CRN module. This tool systematically explores a combinatorial reaction space by iteratively applying a defined set of transformation rules to an initial pool of chemical species (e.g., H_2_O, H^+^). It offers two implementations for this task: a dependency-light, pure Python CRN class and the more computationally efficient MODCRN wrapper for MØD.

CRN’s PathFinder uses A*^36^ to find short synthetic routes. A* needs a conservative (admissible) heuristic to guarantee the shortest paths, but no general chemical admissible heuristic exists, so we use a simple topological distance. This heuristic speeds and biases the search toward shorter routes but can miss alternatives or return topologically valid yet chemically infeasible or impractical steps (e.g., *para*-nitrophenol from benzene; Figure 3D,E). To mitigate this, PathFinder is modular and accepts chemistry-aware scoring functions such as quantum barriers, rule filters, or machine learning (ML) predictors, to rerank, prune, and prioritize candidates toward chemically plausible and experimentally feasible routes.

Alternatively, the MODCRN implementation leverages the high-performance *mod* backend for rapid, exhaustive forward expansion of a predefined chemical space. This approach is better suited for studying the dynamics of closed systems, such as metabolic networks, rather than for open-ended synthesis design, but offers powerful capabilities for visualizing the complete reaction network (Figure S12).

### 3.6 Visualization

The Vis module renders reaction templates as chemically intuitive 2D structures via RDKit conformers, and for abstract forms (DPO rules, large reaction networks), uses MØD and LATEX to generate clear, compact formal graph diagrams (Figure S12).

### 3.7 Case Study

We provide a practical case study to construct a rule library from a reaction database. From 50,016 reactions (USPTO_50k), we aggregate AAMs and use Chem for canonicalization, validation, and deduplication to obtain 44,469 unique reactions. We convert them to ITS graphs with IO, cluster them with Graph to select 336 representatives that are transformed into Rule objects, and apply the rules forward and backward with SynReactor to reconstruct the original molecule graphs with perfect recovery (see Supporting Section H).

## 4 Conclusion

SynKit is an open-source Python toolkit that models reactions as graph transformations, unifying template extraction, canonicalization, and graph clustering on top of RDKit, NetworkX, and optionally MØD. In practice, it groups reactions by shared topologies that frequently align with known mechanistic families and is designed to plug into existing retrosynthesis workflows. By design, this release is topological and omits explicit electronic-structure or condition dependence, and it excludes thermodynamic or kinetic scoring, stereochemical outcomes (regio/chemo/enantioselectivity), and end-to-end route prioritization, so SynKit primarily captures mechanistic patterns rather than feasibility or selectivity.

Current limitations include the lack of a built-in planner or search, no internal ranking for rule-driven product generation, no native yield prediction, limited explicit condition modeling for solvent, temperature, or stoichiometry, graph-based classification that assumes atom–atom maps, and limited stereochemical and regioselectivity operators. We aim to mitigate some of these limitations in future updates as modular extensions. Plans include a scoring API to provide ML or heuristic scorers via plugins, a SynCat^37^ classifier interface for unmapped inputs, a typed-graph layer with stereochemical operators, explicit condition annotations and parsers, optional lightweight quantum-mechanical integration for basic filtering, and planner adapters to integrate with external planners. We will formalize the Mechanistic Transition Graph for branching and recursive pathways with label propagation and implement memory and runtime improvements, e.g., for more efficient canonicalization.^38^

To ensure broad usability, SynKit is pip-installable and exposes a modular API for easy integration and extensibility. Curated documentation and examples, together with built-in provenance tracking, enable transparent, reproducible workflows for academic and industrial adoption.

## Supplementary Material



## Data Availability

The SynKit source code, example data sets, and user documentation are available on GitHub: https://github.com/TieuLongPhan/SynKit. Comprehensive documentation is also hosted at https://synkit.readthedocs.io/en/latest/. All data supporting this study reside in the repository under the Data directory. A permanent archival snapshot of SynKit v1.0.0 (including both code and data) has been deposited on Zenodo (DOI: 10.5281/zenodo.16925060).

## Footnotes

1
MØD is GPL-licensed and may impose redistribution obligations if combined with non-GPL components.

## Notes

Contains detailed methodologies, mathematical explanations, and additional tables and figures that support the manuscript (PDF)

## References

[1] Corey E. J. (1967). General
methods for the construction of complex molecules. Pure and Applied chemistry.

[2] Corey E. J., Long A. K., Rubenstein S. D. (1985). Computer-assisted analysis in organic
synthesis. Science.

[3] Bode, J. W. Reactor, Software Note; ChemAxon Ltd, 2004. https://chemaxon.com/.

[4] Nugmanov R. I., Mukhametgaleev R. N., Akhmetshin T., Gimadiev T. R., Afonina V. A., Madzhidov T. I., Varnek A. (2019). CGRtools: Python library for molecule,
reaction, and condensed graph of reaction processing. J. Chem. Inf. Model..

[5] Landrum, G. , RDKit: A software suite for cheminformatics, computational chemistry, and predictive modeling. 2013; https://www.rdkit.org/.

[6] Coley C. W., Green W. H., Jensen K. F. (2019). RDChiral: An RDKit
wrapper for handling
stereochemistry in retrosynthetic template extraction and application. J. Chem. Inf. Model..

[7] O’Boyle N. M., Banck M., James C. A., Morley C., Vandermeersch T., Hutchison G. R. (2011). Open Babel: An open chemical toolbox. J. Cheminform..

[8] OpenEye Scientific Software, OEChem Toolkit. Software, 2012; https://www.eyesopen.com.

[9] Mahjour B. A., Coley C. W. (2024). RDCanon: a python
package for canonicalizing the order
of tokens in smarts queries. J. Chem. Inf. Model..

[10] Akhmetshin T., Zankov D., Gantzer P., Babadeev D., Pinigina A., Madzhidov T., Varnek A. (2025). SynPlanner: an end-to-end tool for
synthesis planning. J. Chem. Inf. Model..

[11] Genheden S., Thakkar A., Chadimová V., Reymond J.-L., Engkvist O., Bjerrum E. (2020). AiZynthFinder: a fast,
robust and flexible open-source
software for retrosynthetic planning. J. Cheminform..

[12] Blaschke T., Arús-Pous J., Chen H., Margreitter C., Tyrchan C., Engkvist O., Papadopoulos K., Patronov A. (2020). REINVENT 2.0: an AI tool for de novo drug design. J. Chem. Inf. Model..

[13] Cook, M. ; Soloveichik, D. ; Winfree, E. ; Bruck, J. Algorithmic Bioprocesses; Springer: Berlin, Heidelberg, 2009; 543–584.

[14] Andersen J. L., Flamm C., Merkle D., Stadler P. F. (2013). Inferring Chemical
Reaction Patterns Using Graph Grammar Rule Composition. J. Syst. Chem..

[15] Fujita S. (1986). Description
of organic reactions based on imaginary transition structures. 1.
Introduction of new concepts. J. Chem. Inf.
Comput. Sci..

[16] Andersen J. L., Flamm C., Merkle D., Stadler P. F. (2018). Rule composition
in graph transformation models of chemical reactions. MATCH Commun. Math. Comput. Chem..

[17] Andersen, J. L. ; Flamm, C. ; Merkle, D. ; Stadler, P. F. A Software Package for Chemically Inspired Graph Transformation. Graph Transformation: 9th International Conference, ICGT 2016, Held as Part of STAF 2016, Vienna, Austria, July 5–6, 2016, Proceedings; Springer: Cham, 2016; 73–88.

[18] Andersen J. L., Fagerberg R., Flamm C., Fontana W., Kolcak J., Laurent C. V., Merkle D., No̷jgaard N. (2022). Representing
catalytic mechanisms with rule composition. J. Chem. Inf. Model..

[19] Hagberg, A. ; Swart, P. J. ; Schult, D. A. Exploring network structure, dynamics, and function using NetworkX; Los Alamos National Laboratory (LANL), 2007.

[20] Phan T.-L., Weinbauer K., González Laffitte M. E., Pan Y., Merkle D., Andersen J. L., Fagerberg R., Flamm C., Stadler P. F. (2025). SynTemp:
Efficient Extraction of
Graph-Based Reaction Rules from Large-Scale Reaction Databases. J. Chem. Inf. Model..

[21] González
Laffitte M. E., Weinbauer K., Phan T.-L., Beier N., Domschke N., Flamm C., Gatter T., Merkle D., Stadler P. F. (2024). Partial Imaginary Transition State (ITS) Graphs: A
Formal Framework for Research and Analysis of Atom-to-Atom Maps of
Unbalanced Chemical Reactions and Their Completions. Symmetry.

[22] Liu B., Ramsundar B., Kawthekar P., Shi J., Gomes J., Luu Nguyen Q., Ho S., Sloane J., Wender P., Pande V. (2017). Retrosynthetic reaction
prediction using neural sequence-to-sequence
models. ACS central science.

[23] Chen S., An S., Babazade R., Jung Y. (2024). Precise atom-to-atom mapping for
organic reactions via human-in-the-loop machine learning. Nat. Commun..

[24] Schwaller P., Hoover B., Reymond J.-L., Strobelt H., Laino T. (2021). Extraction
of organic chemistry grammar from unsupervised learning of chemical
reactions. Sci. Adv..

[25] McKay B. D., Piperno A. (2014). Practical graph isomorphism, II. Journal of symbolic computation.

[26] Junttila, T. ; Kaski, P. Engineering an efficient canonical labeling tool for large and sparse graphs. 2007 Proceedings of the Ninth Workshop on Algorithm Engineering and Experiments (ALENEX). 2007; 135–149.

[27] Weisfeiler B., Leman A. (1968). The reduction of a graph to canonical form and the algebra which
appears therein. nti, Series.

[28] Shervashidze N., Schweitzer P., van Leeuwen E. J., Mehlhorn K., Borgwardt K. M. (2011). Weisfeiler–Lehman
Graph Kernels. J. Mach. Learn. Res..

[29] Wilcox, C. S. ; Levinson, R. A. In Artificial Intelligence Applications in Chemistry; Pierce, T. H. ; Hohne, B. A. , Eds.; ACS Symposium Series; American Chemical Society: Washington, DC, 1986; 306; 209–230.

[30] Laffitte M. E. G., Beier N., Domschke N., Stadler P. F. (2023). Comparison of Atom
Maps. MATCH Commun. Math. Comput. Chem..

[31] Amigó E., Gonzalo J., Artiles J., Verdejo F. (2009). A comparison
of extrinsic
clustering evaluation metrics based on formal constraints. Information retrieval.

[32] Hubert L., Arabie P. (1985). Comparing partitions. Journal
of classification.

[33] Meilă M. (2007). Comparing
clusterings—an information based distance. Journal of multivariate analysis.

[34] Di
Natale R., Ferro A., Giugno R., Mongiovì M., Pulvirenti A., Shasha D. (2010). Sing: Subgraph search in non-homogeneous
graphs. BMC Bioinformatics.

[35] Corradini, A. ; Montanari, U. ; Rossi, F. ; Ehrig, H. ; Heckel, R. ; Löwe, M. Handbook Of Graph Grammars And Computing By Graph Transformation: Vol. 1: Foundations; World Scientific, 1997; 163–245.

[36] Hart P. E., Nilsson N. J., Raphael B. (1968). A formal basis for the heuristic
determination of minimum cost paths. IEEE transactions
on Systems Science and Cybernetics.

[37] Van Nguyen, P.-C. ; To, V.-T. ; Tran, N.-V. N. ; Phan, T.-L. ; Truong, T. N. ; Gärtner, T. ; Merkle, D. ; Stadler, P. F. SynCat: A Lightweight Graph Neural Network Model to Classify Chemical Reactions. ChemRxiv 2025 10.26434/chemrxiv-2025-5f868, Submitted 2025. This content has not been peer-reviewed.

[38] Andersen J. L., Merkle D. (2020). A Generic Framework for Engineering Graph Canonization
Algorithms. J. Exp. Algorithmics.

