# Supplementary material for: SynKit: A Graph-Based Python Framework for Rule-Based Reaction Modeling and Analysis
Source: J Chem Inf Model. 2025 Dec 3;65(24):13012–9. doi: 10.1021/acs.jcim.5c02123 (PMC12728950; doi:10.1021/acs.jcim.5c02123)
Supplement: Supplementary file 1 [file ci5c02123_si_001.pdf]

Supporting Information

SynKit: A Graph-Based Python Framework for Rule-Based Reaction Modeling and Analysis

Tieu-Long Phan[*,†,‡] Marcos E. González Laffitte[†,¶] Klaus Weinbauer[†,§] Daniel Merkle[‖,‡] Jakob Lykke Andersen[‡] Rolf Fagerberg[‡] Thomas Gatter[†] and Peter F. Stadler[†,⊥,#,@,△,∇]

†*Bioinformatics Group, Department of Computer Science & Interdisciplinary Center for Bioinformatics & School for Embedded and Composite Artificial Intelligence (SECAI), Leipzig University, Härtelstraße 16–18, D-04107 Leipzig, Germany*

‡*Department of Mathematics and Computer Science, University of Southern Denmark, DK-5230 Odense M, Denmark*

¶*Center for Scalable Data Analytics and Artificial Intelligence (ScaDS.AI), Leipzig University, D-04103, Leipzig, Germany*

§*Machine Learning Research Unit, TU Wien Informatics, A-1040 Wien, Austria*

‖*Algorithmic Cheminformatics Group, Faculty of Technology & Center for Biotechnology (CeBiTec), Bielefeld University, Postfach 10 01 31, D-33501, Bielefeld, Germany*

⊥*Max Planck Institute for Mathematics in the Sciences, Inselstraße 22, D-04103, Leipzig, Germany*

#*Department of Theoretical Chemistry, University of Vienna, Währingerstraße 17, A-1090, Vienna, Austria*

@*Facultad de Ciencias, Universidad National de Colombia,Bogotá, Colombia*

△*Center for non-coding RNA in Technology and Health, University of Copenhagen, Ridebanevej 9, DK-1870, Frederiksberg, Denmark*
∇*Santa Fe Institute, 1399 Hyde Park Rd., Santa Fe, NM, 87501, USA*
E-mail: tieu@bioinf.uni-leipzig.de

A Software Overview

SynKit comprises six primary modules, each designed to facilitate specialized tasks within chemical reaction modeling and cheminformatics.

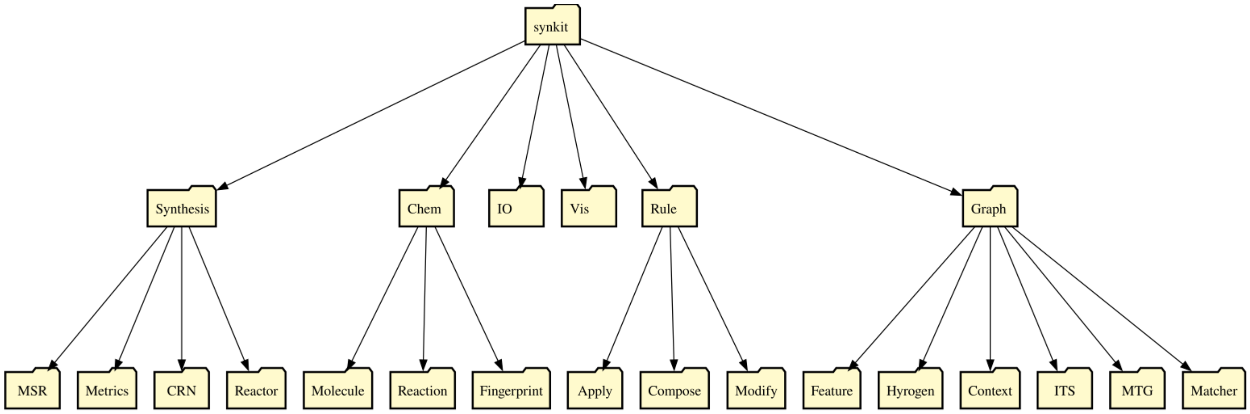

Figure S1: Structural overview of SynKit modules.

SynKit utilizes well-established computational libraries, notably RDKit[1] for cheminformatics functionalities and NetworkX[2] for graph-theoretical operations. All dependencies (including synkit itself) can be installed via PyPI, Conda, or Docker:

Install via PyPI

```
pip install synkit
```

Install via Conda

```
conda install -c tieulongphan synkit
```

Pull Docker image

```
docker pull tieulongphan/synkit:latest
```

Or browse the Docker image on Docker Hub: tieulongphan/synkit.

For comprehensive utilization of SynKit's full capabilities, particularly graph-based reaction modeling employing Double Pushout (DPO) transformation rules, it is recommended to install the MØD software package.[3] The MØD package can be installed either by compiling

directly from source or via a Docker container deployment. Detailed installation instructions for each method are provided at `https://jakobandersen.github.io/mod/installation.html`. For installing in Conda environments the instructions are simply

```
conda install jakobandersen::mod
```

Please note that the Conda does not have a package for a full LaTeX engine, so a separate LaTeX distribution (e.g., TeX Live or MiKTeX) must be installed to enable rendering of DPO rule diagrams. Complete API documentation and tutorials are accessible at `https://synkit.readthedocs.io/en/latest/`.

B Tool Comparison Notes

- SMILES Canonicalization: Only `SynKit` (SMILES/SMARTS) and `RDCanon` (SMARTS only) generate canonical representations that preserve reaction atom-mapping. `CGRTools`, conversely, uses a proprietary signature format to encode these atomic transformations.`RDKit`'s canonicalization is robust for unmapped molecular graphs but struggles to reliably generate a unique representation for complex, atom-mapped reactions.
- Graph Canonicalization: `RDCanon` supports internal graph canonicalization but does not expose it explicitly. `SynPlanner` relies on `CGRTools` for graph canonicalization.
- Atom-map comparison: `SynKit` and `CGRTools` support atom mapping comparison via ITS/CGR isomorphism comparisons.
- ITS/CGR construction: ITS and CGR are equivalent; `CGRTools` and `SynKit` fully supports ITS/CGR.
- Rule clustering: `CGRTools` clusters via partial signature matching; `RDChiral` by exact SMARTS string comparison; and `SynKit` through graph isomorphism.

– Subgraph searching: While `RDKit` is limited to identifying static molecular fragments, both `SynKit` and `CGRTools` (partially) support reaction-aware substructure searching, enabling the identification of specific reaction cores.

– Synthesis Prediction: Predictive capabilities vary: `RDKit` offers basic, single-step forward synthesis. `CGRTools` handles both forward prediction and single-step retrosynthesis, while `SynPlanner` employs a Monte Carlo Tree Search for multi-step pathway elucidation.

– Rule Composition: `SynKit` uniquely enables the construction of complex transformation rules by composing simpler reaction templates, a feature powered by its *nx* and *mod* backends.

– Visualization of reaction schemes relies on the `RDKit` engine for `RDChiral` and `RDCanon`; is absent in `SynPlanner`; and is provided by native, comprehensive modules in `SynKit` and `CGRTool`.

C Mathematical Notation and Preliminaries

This section recapitulates the terminology introduced in previous work on ITS graphs and rule composition.[4,5] For completeness we include here the formalism related to the relevant graph-theoretic notions, the atom-to-atom correspondence underlying balanced reactions, and the construction of *Imaginary transition states* (ITS).[6,7] For fundamental definitions and general terminology on graph theory we refer to the material of F. Harary.[8]

All graphs considered are *finite*, *undirected* and *simple*, i.e., having no loops and without multiple edges. Molecules are modeled by connected graphs whose vertices and edges are, correspondingly, the atoms and bonds in each compound. We therefore consider for every graph G two labeling functions $a_G\colon V(G) \to L_V$ and $b_G\colon E(G) \to L_E$ representing the assignment of atom types and bond orders to vertices and edges of G, respectively. Here L_V

and L_E are understood to be two suitable sets of chemical labels with the only condition of being disjoint, non-empty and without the special symbol $\varnothing \notin L_v \cup L_e$, which we reserve for the construction of the ITS Graph, see Definiton C.1 below.

We should recall, in particular, that two (distinct) vertices $x, y \in V(G)$ are said to be *adjacent* if $uv \in E(G)$. The edge $e = uv \in E(G)$ is then said to be *incident* with u and v.

Now we shall introduce *atom-to-atom maps* and *isomorphisms* between graphs. Both definitions rely on a bijection being established between the vertex sets of two graphs, but since these are, conceptually, two separate ideas, we shall use different notations for each.

Consider first two graphs G and H and a bijection $\alpha\colon V(G) \to V(H)$. We say that α is an *Atom-to-Atom Map (AAM)* between G and H if it preserves vertex labels, i.e., $a_H(\alpha(v)) = a_G(v)$ for all $v \in V(G)$. In particular, it should be noted that whenever G and H model, respectively, the (possibly disconnected) reactants and products graphs of a reaction transforming G into H, denoted by $G \longrightarrow H$, then an AAM exists between G and H if and only if the reaction is *stoichiometrically balanced*.

On the other hand, a function $\varphi\colon V(G) \to V(H)$ is said to be a *morphism* from G to H if it preserves edges, vertex labels and edge labels, i.e., for any pair of vertices $u, v \in V(G)$ it holds that: (i) if $uv \in E(G)$ then $\varphi(u)\varphi(v) \in E(H)$, as well as (ii) $a_H(\varphi(v)) = a_G(v)$ and (iii) $b_H(\varphi(u)\varphi(v)) = b_G(uv)$ whenever $uv \in E(G)$. An injective morphism, moreover, is called a *monomorphism*, while a surjective morphism is referred to as an *epimorphism*. In the special case when φ is a bijective map that also preserves none edges, i.e., $uv \in E(G)$ if and only if $\varphi(u)\varphi(v) \in E(H)$, we say that φ is an *isomorphism* from G to H and write $G \simeq H$. If, additionally, φ is an isomorphism from G to itself, i.e., $\varphi\colon V(G) \to V(G)$, then it is said to be an *automorphism* of G. We note that the set of all automorphisms of a graph G constitutes an algebraic group $Aut(G)$ under the composition of bijections.

The *Imaginary Transition State* (ITS) graph was introduced by Fujita[6] with the purpose of representing reactions in databases. Later it was used by Nugmanov et al[9] for machine learning applications under the name *Condensed Graph of the Reaction* (CGR). It is funda-

mental to the different contributions[4,5,10] we have developed so far. It superimposes reactants and products, encoding bond changes explicitly. Formally it is defined as follows,

Definition C.1. *Let $\alpha\colon V(G) \to V(H)$ be an AAM for a balanced reaction $G \longrightarrow H$. The ITS graph Υ of the triple (G, H, α) has vertex set $V(\Upsilon)$, edge set $E(\Upsilon)$, vertex-labeling function $a_\Upsilon : V(\Upsilon) \to L_V \times L_V$ and edge-labeling function $b_\Upsilon\colon E(\Upsilon) \to (L_E \cup \{\varnothing\}) \times (L_E \cup \{\varnothing\})$, obtained from G by means of a bijection $\tau\colon V(\Upsilon) \to V(G)$ in such a way that*

(i) for all $x, y \in V(\Upsilon)$ we have $xy \in E(\Upsilon)$ iff $\tau(x)\tau(y) \in E(G)$ or $\alpha(\tau(x))\alpha(\tau(y)) \in E(H)$,

(ii) every vertex $x \in V(\Upsilon)$ receives the label $a_\Upsilon(x) = (a_G(\tau(x)), a_H(\alpha(\tau(x))))$,

(iii) every edge $xy \in E(\Upsilon)$ receives the label $b_\Upsilon(xy)$ determined as follows:

$$b_\Upsilon(xy) = \begin{cases} (b_G(\tau(x)\tau(y)), b_H(\alpha(\tau(x))\alpha(\tau(y)))) & \text{if } \tau(x)\tau(y) \in E(G) \text{ and } \alpha(\tau(x))\alpha(\tau(y)) \in E(H) \\ (b_G(\tau(x)\tau(y)), \varnothing) & \text{if } \tau(x)\tau(y) \in E(G) \text{ and } \alpha(\tau(x))\alpha(\tau(y)) \notin E(H) \\ (\varnothing, b_H(\alpha(\tau(x))\alpha(\tau(y)))) & \text{if } \tau(x)\tau(y) \notin E(G) \text{ and } \alpha(\tau(x))\alpha(\tau(y)) \in E(H) \end{cases}$$

When speaking, in particular, of an arbitrary edge uv in an ITS graph, we write $b_\Upsilon(uv) = (b^1_\Upsilon(uv), b^2_\Upsilon(uv))$ to make reference to the diverse values of its label. Then, one last definition worth mentioning is that of the reaction center, which is a special graph constituted by, and only by, the bonds that change during the reaction together with the vertices incident with them. Formally, given the ITS graph $\Upsilon = \Upsilon(G, H, \alpha)$ of a balanced reaction $G \longrightarrow H$ with AAM α, we say that an edge $uv \in E(\Upsilon)$ is a *reaction edge* if $b^1_\Upsilon(uv) \neq b^2_\Upsilon(uv)$. The *reaction centre* is then the subgraph $\Gamma(G, H, \alpha)$ of Υ, induced from Υ by all reaction edges.

D Chemical Conversion Analysis

We evaluated format-conversion performance on the `USPTO_50k` dataset, using a `NetworkX` intermediate for all transformations. Converting from reaction SMILES/SMARTS to `GML` via ITS graph construction required a mean of 2.19 ± 2.15 ms per reaction (Figure S2), with ITS construction alone consuming approximately 75 % of the time (1.58 ± 1.61 ms). Subsequent

extraction of reaction centers and serialization of `GML` encoded rules from the ITS graph was markedly faster, averaging 0.04 ± 0.061 ms and 0.10 ± 0.02 ms per reaction, respectively. In contrast, the reverse pipeline, `GML`→`NetworkX` graph and `NetworkX`→SMILES/SMARTS, which is the most common use case, required 2.83 ± 2.71 ms and 0.92 ± 0.99 ms per reaction, owing to the absence of a direct `GML`→SMILES conversion.

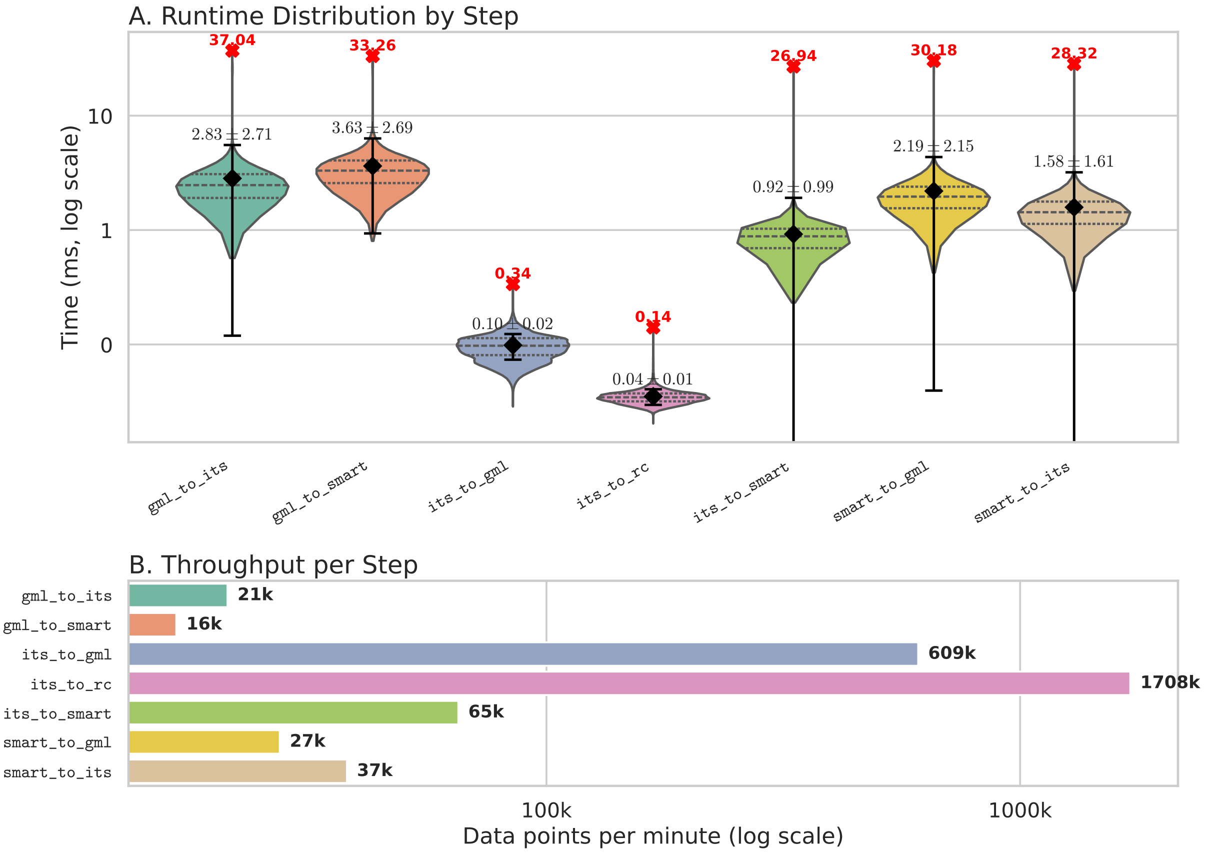

Figure S2: Per-record processing times across conversion types depicted as violin plots (A), and corresponding per-minute throughput for each conversion type (B).

Round-trip checks were performed on the benchmarking dataset (39,732 reactions) for three conversion paths: SMILES ⇄ `ITS`, SMILES ⇄ `GML`, and `GML` ⇄ `ITS`. For each reaction the source representation was canonicalized, converted to the target format and back, canonicalized again, and compared. For `SMILES` a round-trip is successful if the canonicalized SMILES match exactly, including atom-map annotations. For `ITS` and `GML` success is determined by an graph-isomorphism equivalence check that verifies connectivity, atom maps,

formal charges, valence and isotopic labels, and bijective hydrogen counts are equivalent.

For stoichiometrically balanced reactions that include explicit reaction-center hydrogens, the adopted conversion policy yields bijective SMILES $\rightleftarrows$ `ITS` $\rightleftarrows$ `GML` mappings with 100% observed round-trip fidelity. Failures are occur in cases with missing reaction-center hydrogens, non-stoichiometric inputs, atom-map inconsistencies, or tautomer/normalization differences. All modes of failure can be effectively prevented with proper user procedure: preserve explicit hydrogens prior to `GML` export, annotate missing components in unbalanced cases, canonicalize atom maps before comparison, and employ isomorphism-based checks.

E Graph Modeling

E.1 Canonicalization and Approximate Canonicalization

Interface. All implementations receive the same input, a labeled graph $G = (V, E, a, b)$ with label functions $a\colon V(G) \to A \subseteq L_V$ and $b\colon E(G) \to B \subseteq L_E$, for user-defined vertex-label subsets $A \subseteq L_V$ and edge-label subsets $B \subseteq L_E$, and returns the (approximately) canonically relabeled graph denoted by $G^\pi = (V^\pi, E^\pi, a^\pi, b^\pi)$ together with a permutation $\pi\colon V \to \{1, \ldots, |V|\}$ modeling the traversal order established by each algorithm for the vertices of G. The only algorithmic hyper-parameter is the number of refinement iterations $k \in \mathbb{N}$ for `WLGH`; while the `Nauty`- and `Bliss`-inspired algorithm has no tunable parameters. The `Nauty`- and `Bliss`-inspired algorithm inspect the set of labels B; `WLGH` algorithm ignores it.

E.1.1 Algorithmic Details

`Nauty`- and `Bliss`-inspired Algorithm The algorithms refines an initial vertex partition until it is equitable and then performs a depth-first backtracking search over the remaining symmetries.

Given a partition $\Pi = \{C_1, \ldots, C_m\}$ of $V(G)$ and a vertex $v \in V(G)$ we define a labeling

function σ_Π, called signature, as

$$\sigma_\Pi(v) := \Big(a(v),\ \deg(v),\ \big(|N(v) \cap C_i|\big)_{i=1}^{m},\ \mathrm{ms}\{\, b(e)\, \}_{e\in\mathcal{E}(v)}\Big),$$

where $\mathcal{E}(v) := \{\{x, y\} \in E(G) \mid x = v \text{ or } y = v\}$. Two vertices in the same cell receive identical signatures since initially they are taken from the same orbits under the corresponding graph automorphisms.

Below we define the notation used in the pseudocode.

- $\Pi = \{C_1, \ldots, C_m\}$: partition of V into disjoint cells.
- $N(v)$, $\mathcal{E}(v)$: open neighborhood and incident edges of v.
- $\mathrm{ms}(\cdot)$: deterministic multiset→tuple operator (stable sort; canonical float handling).
- $\sigma_\Pi(v)$: signature (refinement key); equal signatures inside a cell induce further splitting.
- When all cells are singletons the leaf permutation is $\mathrm{Flatten}(\Pi)$ and we evaluate $\mathcal{L}(G, \pi)$.
- Working variables: *bestLabel* (current minimal label), *bestPerm* (arg-min permutation).

Algorithmic primitives (used in the pseudocode):

InitPartition group vertices by $\big((a(v)_k)_{k\in A}, \deg(v)\big)$.

Refine repeatedly split each cell into blocks of equal $\sigma_\Pi(v)$ until no further split is possible (equitable partition).

Split individualize $v \in C$: replace C with $\{v\}$ and $C \setminus \{v\}$.

Order deterministic ordering $\textsc{Order}(C) = \mathrm{sort}(C, \mathrm{key} = \kappa)$, where $\kappa(v)$ is e.g. frozen attributes, degree, stable id.

LABEL encode $\pi = (\pi_1, \ldots, \pi_n)$ into the deterministic string $\mathcal{L}(G, \pi) = \text{Node}(\pi) \,\|\|\, \text{Edge}(\pi)$ (node attributes then upper-triangular edge descriptors e_{ij}).

BUILDGRAPH remap $\pi_i \mapsto i$ to produce the canonical graph G^π.

Algorithm S1 GRAPHCANONICALIZATION

Require: Graph $G = (V, E, a, b)$
Require: Vertex keys A, edge keys B
Ensure: Canonically relabelled graph G^π

$\Pi \leftarrow$ INITPARTITION(G, A) ▷ initialise colours
bestLabel $\leftarrow \bot$, *bestPerm* $\leftarrow \bot$
SEARCH$(\Pi, [\,])$ ▷ start search with empty prefix
return BUILDGRAPH$(G, \textit{bestPerm})$

Algorithm S2 SEARCH (depth-first individualization–refinement)

Require: Partition Π of V
Ensure: Updates *bestLabel*, *bestPerm*

$\Pi \leftarrow$ REFINE(Π) ▷ make Π equitable
if $\forall C \in \Pi : |C| = 1$ **then** ▷ reached a leaf (full permutation)
    $\pi \leftarrow$ FLATTEN(Π)
    $\ell \leftarrow$ LABEL(G, π, A, B)
    if $\textit{bestLabel} = \bot \vee \ell < \textit{bestLabel}$ **then**
        bestLabel $\leftarrow \ell$
        bestPerm $\leftarrow \pi$
    end if
    return
end if
$C \leftarrow$ FIRSTNONSINGLETON(Π)
for all $v \in$ ORDER(C) **do** ▷ deterministic order via $\kappa(v)$
    $\Pi' \leftarrow$ SPLIT(Π, C, v) ▷ individualize v
    SEARCH(Π')
end for

Implementation note. The pseudocode above shows the basic individualization–refinement (IR) routine with depth-first exploration. The full implementation (beyond this basic pseudocode) also supports: automorphism pruning with orbit computation,[11] pruning via partial labels (partial certificates used to lexicographically prune branches),[12] and an optional recursion cap to guarantee termination on very large instances (though then no result or a non-canonical result may be returned).

`WLGH` **approximate canonicalization algorithm.** Here, we implement an algorithm for approximating canonicalization based on the Weisfeiler-Lehman graph hashing algorithm based on `NetworkX` (`networkx.algorithm.weisfeiler_lehman_subgraph_hashes`). The approximation stems from the fact that the graph returned is not guaranteed to be canonical. Define $\mathrm{ms}(\cdot)$ as a deterministic multiset-tuple operator that returns neighbor labels as a lexicographically sorted tuple with preserved/summed multiplicity). If edges carry labels (bond order, aromaticity, etc.) include the incident edge label in each neighbour entry, e.g. $(\ell_{(v,u)}, \lambda^{(t)}(u))$. Then

$$\begin{aligned}
\lambda^{(0)}(v) &= a(v), \\
\lambda^{(t+1)}(v) &= \mathrm{hash}\Big(\lambda^{(t)}(v),\ \mathrm{ms}\{\lambda^{(t)}(u)\}_{u \in N(v)}\Big), \qquad 0 \le t < k, \\
\kappa(v) &= \big(\lambda^{(k)}(v),\ \deg(v)\big).
\end{aligned}$$

Obtain the canonical ordering π by a stable lexicographic sort of V with key κ,

$$\pi := \mathrm{argsort}\big(V;\ \kappa\big),$$

and construct the explicit remapping $\mathrm{old2new} : V \to \{1, \ldots, |V|\}$ by assigning new indices in π-order (i.e. $\mathrm{old2new}(\pi[i]) = i$). Build $G^\pi = (V^\pi, E^\pi)$ by creating vertices $1, \ldots, |V|$ with the corresponding attributes and remapping edges

$$E^\pi = \{(\mathrm{old2new}(u), \mathrm{old2new}(w), \mathrm{attr})\ :\ (u, w, \mathrm{attr}) \in E\}.$$

Lines 1 through 8 is implemented in `networkx.algorithm.weisfeiler_lehman_subgraph_hashes` while the remaining part is our implementation of the approximate canonicalization.

Algorithm S3 WLGH

Require: Graph $G = (V, E, a, b)$, vertex keys $A \subseteq L_V$, number of iterations $k \in \mathbb{N}$
Ensure: Canonically relabeled graph G^π

```
for all v ∈ V do
    λ^(0)(v) ← a(v)
end for
for t = 0 to k − 1 do
    for all v ∈ V do
        λ^(t+1)(v) ← hash(λ^(t)(v), ms{λ^(t)(u)}_{u∈N(v)})
    end for
end for
κ(v) ← (λ^(k)(v), deg(v))
π := sort(V, κ)
for i = 1 to |V| do
    get v := V(G)[i] and add v to V(G^π) with index π(v)
end for
build G^π using V(G^π) and π
return G^π
```

E.1.2 Performance evaluation

We evaluated the correctness of our algorithms on the `USPTO_3K` dataset.[4,13] Using `SynTemp` we compared the mapper pairs (`RXNMapper`, `LocalMapper`) and (`GraphormerMapper`, `LocalMapper`). For a mapper pair (A, B) of the same reaction i (when both mappers produced outputs) denote:

$M_i^{(A)}, M_i^{(B)}$ — mapped molecular graphs from mappers A and B,

$S_i^{(A)}, S_i^{(B)}$ — canonicalizer string outputs for the same RSMI but from different mappers.

The adjudicator (`SynTemp` isomorphism) and canonicalizer indicators are

$$I_i^{(A,B)} = \mathbf{1}\{M_i^{(A)} \cong M_i^{(B)}\} \quad (\texttt{SynTemp}\ \text{isomorphism}), \qquad C_i^{(A,B)} = \mathbf{1}\{S_i^{(A)} = S_i^{(B)}\}.$$

Let $N_{ab}^{(A,B)} = \#\{i :\ I_i^{(A,B)} = a,\ C_i^{(A,B)} = b\}$ for $a, b \in \{0, 1\}$ and $N^{(A,B)} = \sum_{a,b} N_{ab}^{(A,B)}$.

Agreement (Concordance). We report the per-pair agreement (concordance) as

$$\text{Agreement}^{(A,B)} = \frac{N_{11}^{(A,B)} + N_{00}^{(A,B)}}{N^{(A,B)}}.$$

Table S1: Interpretation of outcome pairs (I, C) where I is the `SynTemp` isomorphism indicator and C is the canonicalizer-equality indicator.

(I, C)	Short label	Meaning
$(1, 1)$	*Agree / True–True*	Mapped graphs are isomorphic and canonicalizer strings match — expected / correct. Report $N_{11}^{(A,B)}$.
$(0, 0)$	*Agree / False–False*	Mapped graphs are non-isomorphic and canonicalizer strings differ — expected / correct. Report $N_{00}^{(A,B)}$.
$(0, 1)$	*Collision*	Graphs are non-isomorphic but canonicalizer produced identical strings — indicates a canonicalizer collision.
$(1, 0)$	*Failure*	Graphs are isomorphic yet canonicalizer strings differ — indicates a canonicalizer failure.

In our implementation we observed no canonicalizer failures: $N_{10}^{(A,B)} = 0$ for both mapper pairs (i.e., no *Failure* cases recorded).

Table S2: Exact-match canonicalization agreement against `SynTemp` graph isomorphism.

**Algorithm**	**Agreement**	
	`RXNMapper` vs `LocalMapper`	`GraphormerMapper` vs `LocalMapper`
$\texttt{WLGH}_1$	0.705	0.619
$\texttt{WLGH}_2$	0.952	0.900
$\texttt{WLGH}_3$	0.991	0.946
`Nauty`- and `Bliss`-inspired	1.000	1.000
`CGRTools`	0.990	0.980
$\texttt{RDCanon}_{\text{all}}$	0.851	0.857
$\texttt{RDCanon}_{\text{success\_case}}$	0.913	0.922

Furthermore, we benchmarked runtime performance on 39,732 atom-mapped reactions drawn from `USPTO_50K` via `SynTemp`. Violin plots of per-reaction canonicalization times are shown in Figure S3, and pairwise statistical comparisons appear in Figure S4. While the `Nauty`- and `Bliss`-inspired algorithm achieves a 100 % agreement with isomorphism comparison, it is orders of magnitude slower than `WLGH` ($p < 0.05$, see Figure S3A and Figure S4A). In contrast, the $\texttt{WLGH}_2$ and $\texttt{WLGH}_3$ variants process graphs in approximately 1 ms while maintaining agreement rates above 90 %.

Benchmarking revealed significant limitations in `RDChiral`, including frequent *canonical-*

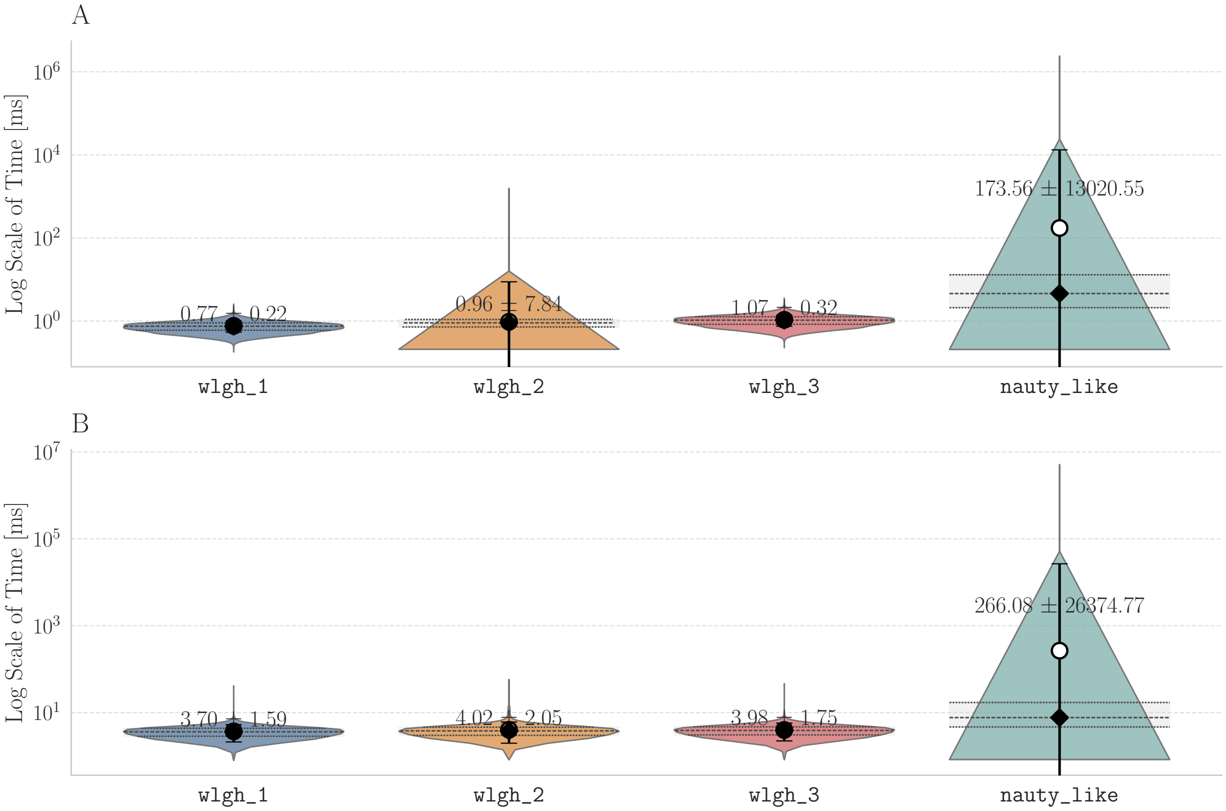

Figure S3: Per-record processing times for graph canonicalization methods, shown as violin plots. (**A**) ITS-graph canonicalization with `GraphCanonicaliser`. (**B**) Reaction SMILES with atom maps canonicalization using `CanonRSMI`. Methods compared: $\texttt{WLGH}_{1-3}$-like and `Nauty`- and `Bliss`-inspired algorithms.

ization failures and *memory leaks* that preclude its use in large-scale applications. When tested on the `USPTO_3K` dataset, it failed to process numerous reaction SMARTS derived from `RXNMapper` (179), `GraphormerMapper` (188), and `LocalMapper` (198). Furthermore, its performance was inefficient, with an average processing time of 0.5 ± 3.4 s per reaction, markedly slower than the `Nauty`- and `Bliss`-inspired or $\texttt{WLGH}_3$ algorithms. Critically, even after excluding these canonicalization failures, its agreement remained inferior to both `Nauty`-`Bliss`-inspired and $\texttt{WLGH}_3$ algorithm (Table S2).

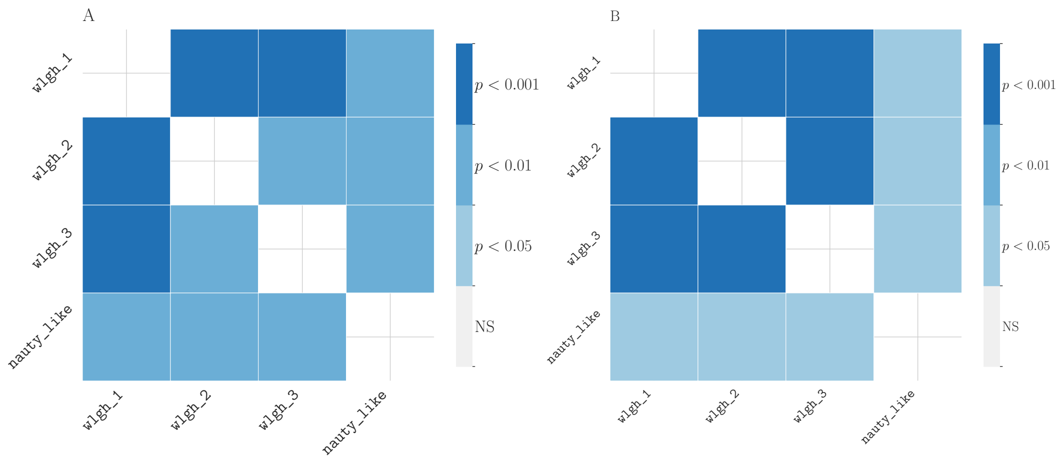

Figure S4: Holm-corrected paired t-test heatmaps of canonicalization runtimes: (**A**) ITS-graph via `GraphCanonicaliser`; (**B**) Reaction SMILES via atom-map-aware `CanonRSMI`. Significance: NS, $p < 0.05$, $p < 0.01$, $p < 0.001$.

E.2 Mechanistic Transition State Graph MTG

E.2.1 Implementation Note

The mechanistic transition state graph (MTG) is designed to summarize an overall reaction for which a sequence of intermediates and their connecting atom-atom-maps are known or have been computed. The atom-atom-maps are conveniently encoded as ITS graphs describing the transitions between consecutive step. Assume k chronological intermediates

$\Upsilon_0, \ldots, \Upsilon_{k-1}$ (notation and symbols are defined in detail in Section C.) and $k-1$ precomputed node maps M_i : nodes$(\Upsilon_i) \rightharpoonup$ nodes(Υ_{i+1}), $i = 0, \ldots, k-2$, one for each step. Together with M_i, Υ_{i-1} and Υ_i define the ITS graphs for a single step. The MTG is now obtained by superimposing all Υ_i along the maps M_i. This idea of superimposing is similar to the recent *overlay graph* framework,[14] but the MTG concept differs by encoding also the temporal order of the steps, as we describe now.

In the MTG, each node and edge is annotated with a *variable-length tuple* attribute; in particular the edge attribute `order` is a tuple that may contain the absence symbol $\varnothing$. Node histories are formed identically to edge histories; we therefore state the rules only for edges. For a frame sequence $\Upsilon = \langle \Upsilon_0, \ldots, \Upsilon_{k-1} \rangle$, the length-$k$ framewise attribute history of an element κ is denoted by $\mathcal{H}_\kappa[0{:}k-1]$ and is obtained by applying the local transition rules A, B, and C given below to each adjacent pair $(\Upsilon_i, \Upsilon_{i+1})$ for $i = 0, \ldots, k-2$. If the reader prefers to omit the formalism that follows, an illustrative history is provided in Section E.2.2, based on the often occuring case that edge labels are bond order values.

Denote by $O_i(u, v)$ the MTG tuple for edge (u, v) in frame Υ_i ($\varnothing$ if absent). We write O_i when (u, v) is clear, and write $O_i = (a, b)$ to assign bond-order attributes. Propagate mappings M_i forward from Υ_0 to obtain a product id (pid) for each local node; each local edge then has the canonical undirected pid pair κ. Apply the following local rules to each adjacent pair $(\Upsilon_i, \Upsilon_{i+1})$:

(A) Tuples present in both frames. Let $O_i = (a, b)$ and $O_{i+1} = (b, c)$ (so $\mathrm{tail}(O_i) = \mathrm{head}(O_{i+1})$). If $\mathrm{tail}(O_i) \neq \mathrm{head}(O_{i+1})$ flag an error. Set $\mathcal{H}_\kappa[i] \coloneqq O_i$, $\mathcal{H}_\kappa[i+1] \coloneqq O_{i+1}$ and concatenate the suffix of O_{i+1} after its head to yield (a, b, c).

(B) Carry-forward (tuple present only in Υ_i). Let $O_i = (a, b)$ and $O_{i+1} = \varnothing$; then set $\mathcal{H}_\kappa[i] \coloneqq O_i$, $\mathcal{H}_\kappa[i+1] \coloneqq \big(\mathrm{tail}(O_i), \mathrm{tail}(O_i)\big)$, which satisfies (A) and yields (a, b, b). More generally, if $O_t = (a, b)$ and $O_{t+1} = \cdots = O_{t+n} = \varnothing$, propagation produces the per-frame tuple $(a, \underbrace{b, \ldots, b}_{n+1})$; if $O_{t+n+1} = (b, c)$ concatenation then yields $(a, \underbrace{b, \ldots, b}_{n+1}, c)$.

(C) Carry-backward (tuple present only in Υ_{i+1}). Let $O_i = \varnothing$ and $O_{i+1} = (b, c)$. Then set $\mathcal{H}_\kappa[i] \coloneqq (\text{head}(O_{i+1}), \text{head}(O_{i+1}))$, $\mathcal{H}_\kappa[i+1] \coloneqq O_{i+1}$, which satisfies (A) and concatenates to yield (b, b, c). More generally, if $O_{t-n} = \cdots = O_{t-1} = \varnothing$ and $O_t = (b, c)$, propagation yields the per-frame tuple $(\underbrace{b, \ldots, b}_{n}, c)$.

E.2.2 Example and Analysis

Figure S5 shows a two-step aldol addition used to illustrate MTG construction. Applying rules (A)–(C) to every adjacent frame pair produces the per-edge, framewise tuples quoted below; we give each example together with the specific rule applications that produce it.

- $(2, 1, 2)$ — a C-O bond: double, then single, then double. Here $O_0 = (2, 1)$, $O_1 = (1, 2)$; hence $\mathcal{H}_\kappa = ((2, 1), (1, 2)) = (2, 1, 2)$.
- $(1, \varnothing, \varnothing)$ — a C-H bond: single, broken, then remains broken. Given $O_0 = (1, \varnothing)$, $O_1 = \varnothing$, applying (B) to (Υ_0, Υ_1) yields $\mathcal{H}_\kappa[0] = (1, \varnothing)$, $\mathcal{H}_\kappa[1] = (\varnothing, \varnothing)$; hence $\mathcal{H}_\kappa = ((1, \varnothing), (\varnothing, \varnothing)) = (1, \varnothing, \varnothing)$.
- $(2, 2, 1)$ — a C-O bond: double, double, then single. Given $O_0 = \varnothing$, $O_1 = (2, 1)$, applying (C) to (Υ_0, Υ_1) sets $\mathcal{H}_\kappa[0] = (2, 2)$, $\mathcal{H}_\kappa[1] = (2, 1)$; hence $\mathcal{H}_\kappa = ((2, 2), (2, 1)) = (2, 2, 1)$.

Future work will render long or branching sequences using compression and focus-context techniques, event aggregation, selective expansion, and an interactive viewer that will reveal full per-step tuple vectors on demand. For mechanisms with ambiguous event ordering, we will extend the MTG to support partial orders or equivalence classes of histories.

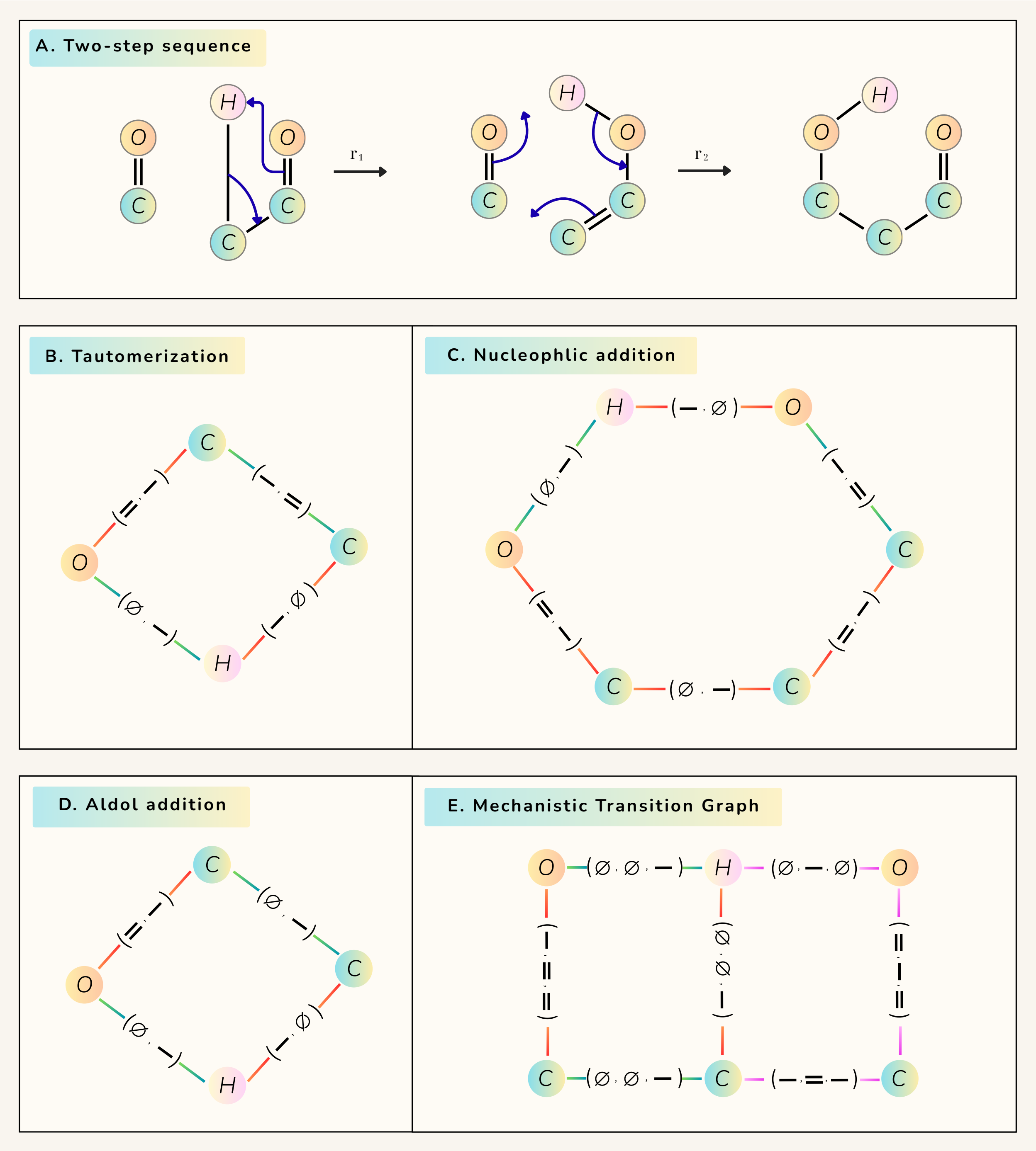

Figure S5: MTG example for a two-step aldol addition reaction: (A) overall sequence; (B) step-1 center (tautomerization); (C) step-2 center (nucleophilic addition); (D) net reaction center before water elimination; (E) resulting Mechanistic Transition Graph.

E.3 Graph Matcher

E.3.1 Graph Clustering

Let $\mathcal{S} = \{Q_r^{(i_1)}, \ldots, Q_r^{(i_L)}\}$ be the set of reaction-template graphs extracted at expansion radius r from L distinct ITS graphs. Algorithm S4 partitions $\mathcal{S}$ into isomorphism classes by combining a Weisfeiler-Lehman graph hash with an exact VF2 isomorphism test (`networkx.algorithm.weisfeiler_lehman_graph_hashes`). The graph hash serves as an inexpensive equivalence filter: two templates are subjected to VF2 only if their hashes coincide.

Algorithm S4 GraphCluster$_r$: hash-filtered agglomerative clustering of templates

Require: Set $\mathcal{S} = \{Q_1, \ldots, Q_L\}$ of $L \geq 2$ graphs
Ensure: Partition $\mathcal{T}$ of $\mathcal{S}$ into isomorphism classes

```
 1: H ← {Q ↦ GraphHash(Q) : Q ∈ S}                ▷ pre-compute graph hashes
 2: T ← ∅
 3: while S ≠ ∅ do
 4:     Select and remove an arbitrary template Q* ∈ S
 5:     C ← {Q*}                                    ▷ current cluster
 6:     for all Q ∈ S with H[Q] = H[Q*] do
 7:         if VF2_Isomorphic(Q, Q*) then
 8:             C ← C ∪ {Q};   S ← S \ {Q}
 9:         end if
10:     end for
11:     T ← T ∪ {C}
12: end while
13: return T
```

Algorithm S5 extends GraphCluster to data streams or corpora that cannot be loaded entirely into memory. Incoming records are processed in batches of predefined fixed size b_0, each batch being clustered against the current template repository $\mathcal{T}_{\text{upd}}$. The template list is incrementally augmented with one representative per newly discovered isomorphism class, so that subsequent batches benefit from an ever more comprehensive hash dictionary.

Algorithm S5 BatchCluster($\mathcal{D}, \mathcal{T}_0, b_0$): scalable, state-ful template clustering

Require: $\mathcal{D} = \{D_1, \dots, D_N\}$ ▷ reaction records with ITS templates
$\mathcal{T}_0$ ▷ initial template repository (possibly empty)
$b_0 \in \mathbb{N}^+$ ▷ batch size
Ensure: processed records $\mathcal{D}_{\text{proc}}$ with cluster labels,
updated template repository $\mathcal{T}_{\text{upd}}$
$\mathcal{T}_{\text{upd}} \leftarrow \mathcal{T}_0$; $\mathcal{D}_{\text{proc}} \leftarrow \varnothing$
for all batches $\mathcal{B} \subseteq \mathcal{D}$ of size b_0 **do**
    Extract template set $\mathcal{S} \leftarrow \{Q \mid Q \in \mathcal{B}\}$
    $(\mathcal{C}_{\text{batch}}, \mathcal{T}_{\text{upd}}) \leftarrow \text{GraphCluster}_r(\mathcal{S} \cup \mathcal{T}_{\text{upd}})$
    Annotate $\mathcal{B}$ with cluster labels $\mathcal{C}_{\text{batch}}$
    $\mathcal{D}_{\text{proc}} \leftarrow \mathcal{D}_{\text{proc}} \cup \mathcal{B}$
end for
return $(\mathcal{D}_{\text{proc}}, \mathcal{T}_{\text{upd}})$

Scalability. Memory consumption is bounded by $b_0 + |\mathcal{T}_{\text{upd}}|$ graphs, and the amortized runtime per incoming template is dominated by one graph hash and, in the occasional event of a collision, a single VF2 call. Consequently, BatchCluster supports continuous ingestion of millions of reaction templates on commodity hardware.

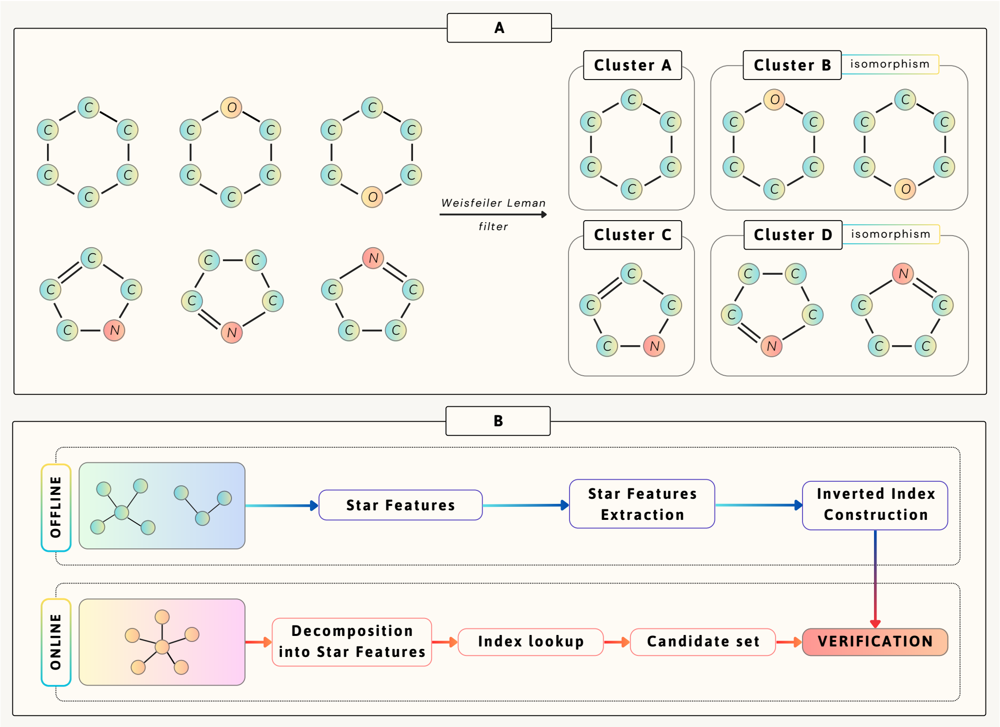

Figure S6: (A) Graph clustering via `WL` full graph hashing. (B) Substructure search via `SING` with offline feature indexing.

Runtime analysis We performed two benchmarking experiments to evaluate the wall-clock runtime and scalability of our clustering implementations using the `nx` and `mod` backends. Each configuration was executed in triplicate; we report the median runtime and interquartile range (IQR).

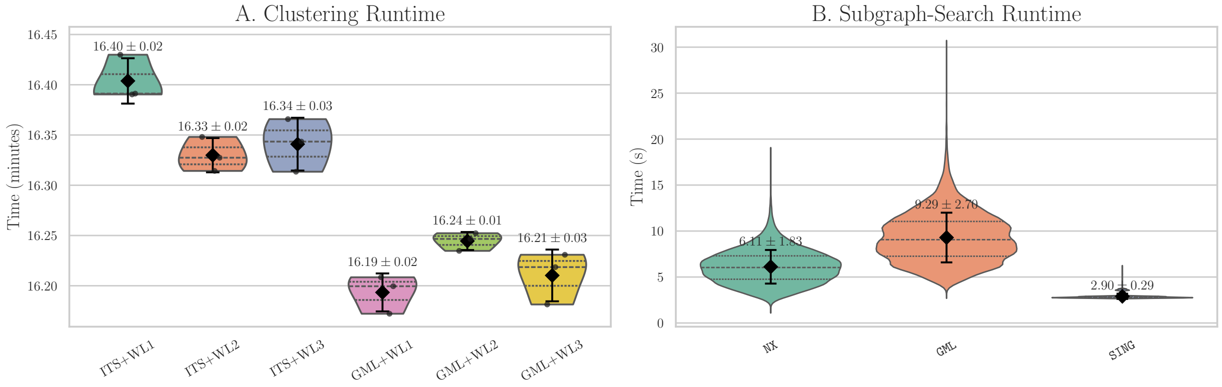

Figure S7: Comparison of algorithm runtimes: (A) Graph clustering across graph hash configurations using different backends. (B) Subgraph search across various backends and query algorithms.

Here, for brevity, $\texttt{WL}_n$ denotes the Weisfeiler Lehman graph-hashing algorithm with n iterations Figure S7A shows that employing graph hash pre-filters ($\texttt{WL}_2$, $\texttt{WL}_3$) reduces the number of full isomorphism tests and thus accelerates clustering substantially compared to the unfiltered (`generic`) approach. Across all configurations, the `mod` backend outperforms `nx` by an order of magnitude. Furthermore, $\texttt{WL}_2$ and $\texttt{WL}_3$ exhibit comparable runtimes, indicating diminishing returns beyond two refinement iterations.

Clustering quality by WL iteration A `WL` prefilter does not alter the final clustering outcome because exact VF2 isomorphism checks are still performed within each WL bucket. The prefilter was assessed on the benchmarking dataset (39,732 reactions) by comparing WL-only bucketings to the isomorphism-based clustering using three standard measures: purity,[15] the adjusted Rand index (ARI),[16] and normalized mutual information (NMI).[17] Results are summarised in Table S3 and illustrated in Fig. S8.

Clustering quality improves monotonically with WL iteration L. The WL-only diagnostic

Table S3: Clustering metrics by WL iteration.

WL iteration	Purity	ARI	NMI
1	0.991 493	0.629 370	0.999 425
2	0.999 773	0.985 915	0.999 985
3	0.999 975	0.998 415	0.999 998
4	1.000 000	1.000 000	1.000 000
5	1.000 000	1.000 000	1.000 000

demonstrates that, on this dataset, increasing L steadily closes the gap to the VF2 baseline; by $L = 4$ the WL partitioning attains perfect agreement with VF2 (purity = ARI = NMI = 1.0), and no further improvement is observed at $L = 5$. These results support using WL as a fast prefilter: it substantially reduces the number of expensive VF2 comparisons while preserving clustering fidelity.

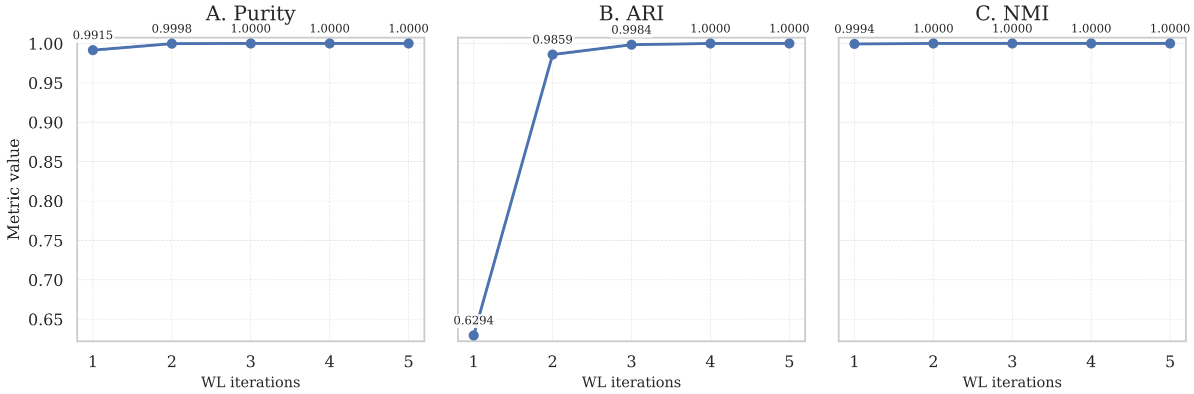

Figure S8: Clustering quality vs WL iteration (1–5). Panels: (A) Purity, (B) ARI, (C) NMI. All metrics improve with depth: Purity and NMI reach 1.00 by $L = 4$, while ARI rises from 0.63 to 1.00, indicating near-perfect agreement with VF2.

E.3.2 Subgraph Search

Here, SynKit extracted 270 reaction rules from the benchmarking database. For comparison, we also evaluated RDChiral[18] in template extraction, which yielded 10,065 reaction templates prior to deduplication. Since RDChiral lacks canonicalization, we applied our own canonicalization procedure, resulting in 1,892 unique templates. The reaction rules gener-

ated by SynKit are more fine-grained and provide broader coverage of the reaction space. We note, however, that RDChiral currently handles stereochemistry more effectively, a feature planned for future SynKit releases.

Figure S7B confirms that both backends scale linearly with batch size. In practice, since $\texttt{WL}_3$ is already computed during canonicalization, its fingerprint can be reused directly—eliminating redundant hashing—and thus serving as the default for production-grade clustering. The SING[19] algorithm is a fast "filter-and-refine" method for finding all occurrences of a small query graph within a much larger target graph. First, it creates a unique "fingerprint" for each node in both graphs by cataloging the sequences of labels along short walks starting from that node. It then uses these fingerprints to quickly filter the massive target graph, identifying a small set of candidate nodes for each node in the query graph. Only nodes that share the exact same fingerprint profile are considered potential matches. Finally, it performs an intelligent search on these much smaller candidate lists to verify the connections and piece together the complete matching subgraphs, avoiding a slow, brute-force search across the entire target graph.

F Rule module

This section reports a controlled study quantifying how the template expansion radius affects both retrieval quality and computational cost for a representative transformation. We selected N-alkylation (fluoride) and curated $N = 553$ reactions sharing an identical reaction center. The set was split once into training $\mathcal{D}_{\text{train}}$ and held-out test $\mathcal{D}_{\text{test}}$ with a fixed seed (seed=42). Starting at the reaction center ($r = 0$), templates learned on $\mathcal{D}_{\text{train}}$ were expanded by graph radius $r \in \{0, 1, 2, 3, 4, 5\}$. For each radius we (i) applied the resulting template set to $\mathcal{D}_{\text{test}}$ and measured standard retrieval metrics, and (ii) recorded wall-clock time for rule application. The column n in Table S4 is the number of distinct (deduplicated) template variants at radius r.

We adopt a per-reaction retrieval view. Let $N = |\mathcal{D}_{\text{test}}|$ and index test reactions by $i = 1, \ldots, N$. Denote the ground truth by g_i and the set of predicted products by P_i.

$$\text{Recall} \;=\; \frac{1}{N}\sum_{i=1}^{N} \mathbf{1}\big[g_i \in P_i\big], \qquad \text{prec}_i \;=\; \begin{cases} \frac{|\{p\in P_i:\, p=g_i\}|}{|P_i|}, & |P_i| > 0, \\ 0, & |P_i| = 0, \end{cases} \qquad \text{Precision} \;=\; \frac{1}{N}\sum_{i=1}^{N} \text{prec}_i,$$

$$\text{F}_1 \;=\; 2\,\frac{\text{Precision}\cdot\text{Recall}}{\text{Precision}+\text{Recall}}.$$

Table S4: Effect of template radius on rule application: number of unique templates n, wall-clock time (s) for application, and retrieval metrics on $\mathcal{D}_{\text{test}}$.

r	n	Time (s)	Recall	Precision	F_1
0	1	1.79	1.000	0.624	0.769
1	22	5.51	0.982	0.657	0.787
2	184	41.75	0.946	0.674	0.787
3	363	76.73	0.766	0.562	0.648
4	407	82.66	0.396	0.372	0.384
5	429	88.20	0.252	0.239	0.245

The results exhibit a clear precision-recall trade-off, which is clearly illustrated in Figure S9. At $r = 0$ (reaction center only), recall is maximal (permissive templates) but precision is limited. Increasing r increases specificity and n, which raises matching time and, beyond a point, harms recall. On this dataset the best balance occurs at $r = 1$–2: F_1 is identical (0.787) at $r = 1$ and $r = 2$, while $r = 1$ is markedly faster (5.51 s vs. 41.75 s; Table S4). To balance effectiveness and throughput in the rule-refinement pipeline, $r = 1$ is adopted as the default expansion radius.

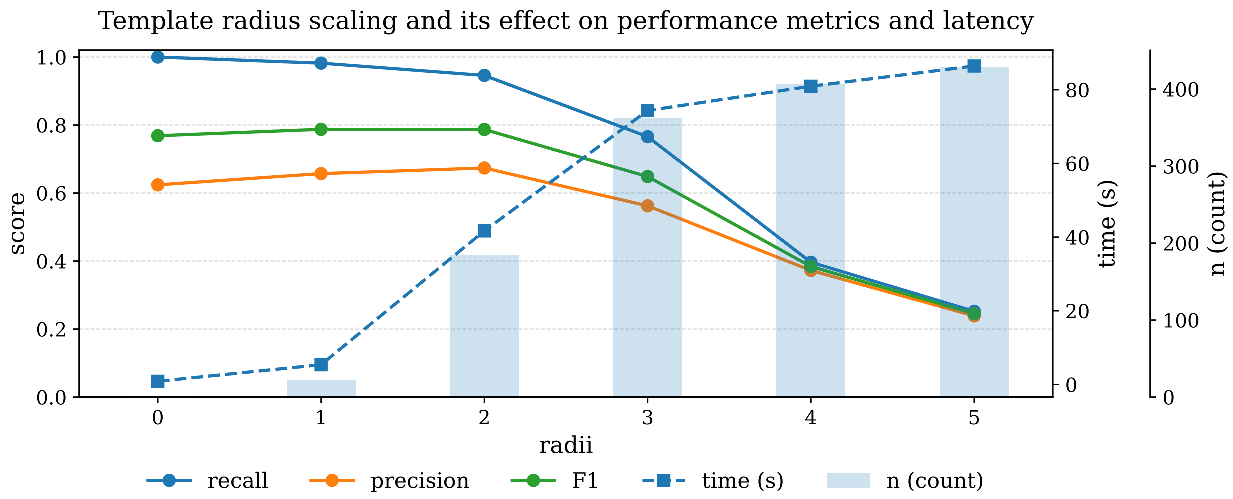

Figure S9: Performance and cost versus template radius r. Plotted are Recall, Precision and F_1 (left axis), wall-clock time (right axis), and unique template count n (inset).

G Synthesis

G.1 Reactor

This section presents a formal comparison of computational behaviour across `Reactor` configurations in `SynKit`. The `Reactor` API implements DPO graph transformations:[20] a reaction rule is specified by the span $(L \xleftarrow{l} K \xrightarrow{r} R)$. For a substrate graph G and a match $m\colon L \to G$, two pushout constructions are made to the product graph H. The `Reactor` workflow decomposes into two subtasks: subgraph search (pattern matching) and graph transformation. We compare two backends. `MODReactor` (*mod*) is a thin wrapper exposing the native `MØD` C++ DPO engine and therefore delegates matching and pushout computations to the native implementation; `SynReactor` (*nx*) implements a DPO-like transformation on top of `NetworkX`, emits ITS-style graph objects instead of the raw product graph H.

To limit combinatorial explosion during matching we implement chemically informed strategies. The *COMPONENT* mode constrains matches to be intermolecular when multiple connected components are present (capturing a natural reagent/substrate partition), *ALL* permits both intra- and intermolecular matches (unconstrained), and *BT* (bimodal technique or hybrid) performs an exhaustive fallback (*ALL*) search when simpler (*COMPONENT*) strategies fail. Empirically, enforcing *COMPONENT*, a lightweight chemical prior, substantially prunes the search space and yields typical speedups of $\approx 1.5\times$ in mean execution time relative to *ALL* (see Figure S10). This is particularly effective for large templates and multi-component reactions (e.g., bimolecular processes, protecting-group contexts) where many embeddings are chemically irrelevant.

Architectural differences further affect runtime and memory trade-offs. *nx* uses an implicit (attributed) hydrogen model (hydrogen counts stored on heavy-atom vertices) and produces ITS-native outputs convenient for template-driven analyses (isotope tracing, mechanism propagation). By contrast, *mod* operates on explicit hydrogenated graphs and returns the raw product graph H, which downstream tools may require. Although the C++ backend

(*mod*) typically offers lower per-operation overhead and higher raw throughput. Conversely, *nx*'s implicit-hydrogen representation in patterns reduces isomorphism work and can yield faster end-to-end discovery for ITS-centric workflows despite Python's higher per-operation cost. In practice, choose *mod* for throughput-sensitive, large-scale batch processing that demands explicit graphs, and *nx* for prototyping, ITS-native pipelines, or when chemically-aware match constraints (e.g., *COMPONENT*) are applicable. All timing and memory comparisons reported here use identical rule sets and substrates; see Figure S10 for quantitative results.

Therefore, users may tune `Reactor` along three axes—(1) backend: *mod* (C++) or *nx* (`NetworkX`/Python); (2) matching strategy: *COMPONENT*, *ALL*, or *BT*; and (3) hydrogen handling: *explicit* or *implicit*—to balance throughput, memory usage, and ITS-native expressivity. Note that the *mod* backend supports only *explicit* hydrogen handling.

For high-throughput screening (HTS), the search protocol was augmented with a pre-screening step to rapidly filter candidate molecules against multiple reaction patterns. We implemented the `SING`[19] algorithm for this purpose, which accelerated the default *nx* backend by a factor of approximately 1.8 (see Figure S11). Despite this significant optimization, the raw computational speed of the C++-based *mod* backend remained the superior performer in these large-scale scenarios.end.

G.2 Chemical Reaction Network

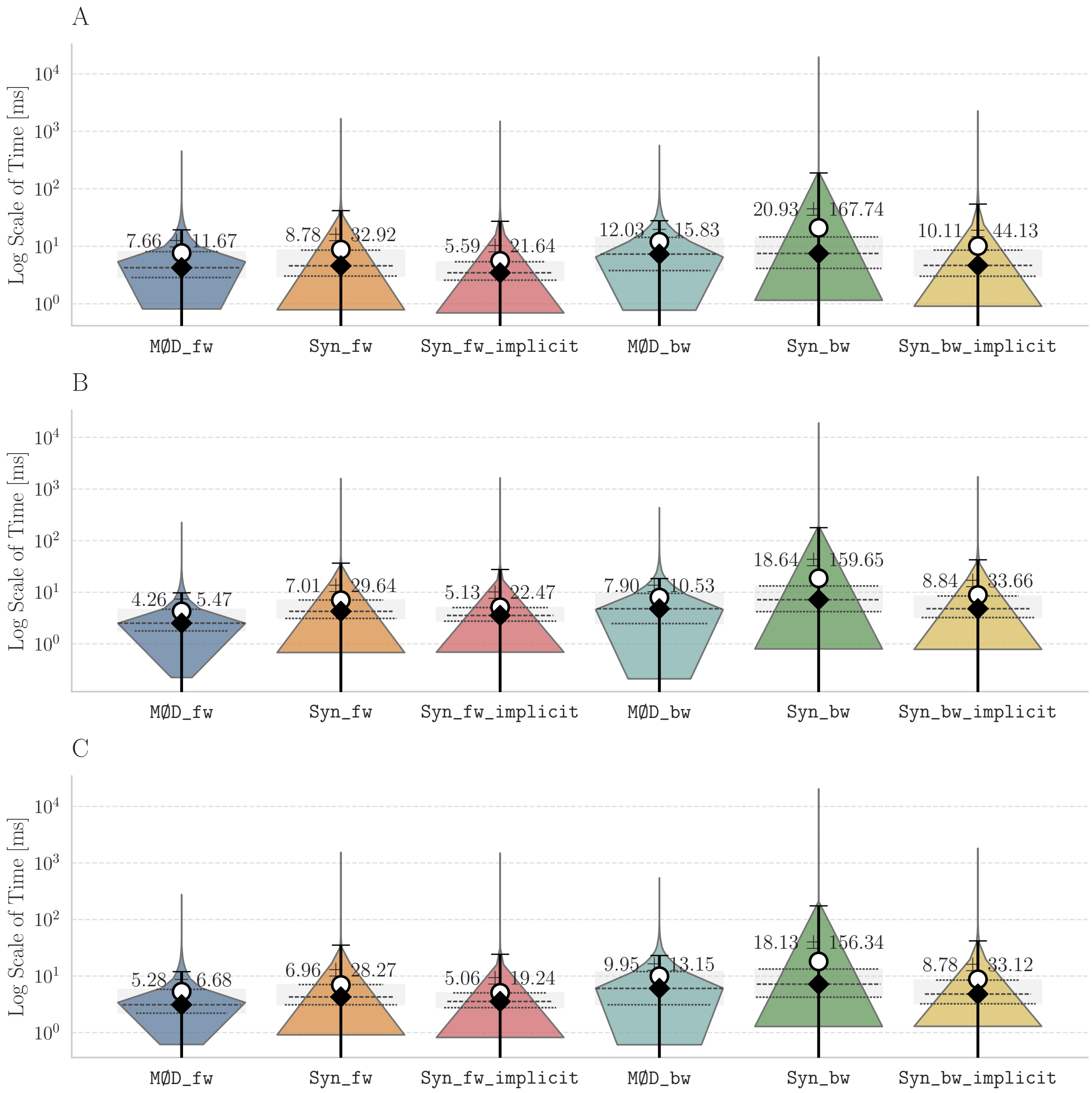

Figure S10: Comparison of run times for three configurations: the *mod* backend, and the *nx* backend with explicit versus implicit hydrogen handling in both forward and backward reactions. Panels (A), (B), and (C) correspond to the three search strategies for identifying reaction patterns within a host molecule: **ALL**, **COMPONENT**, and **BT**, respectively.

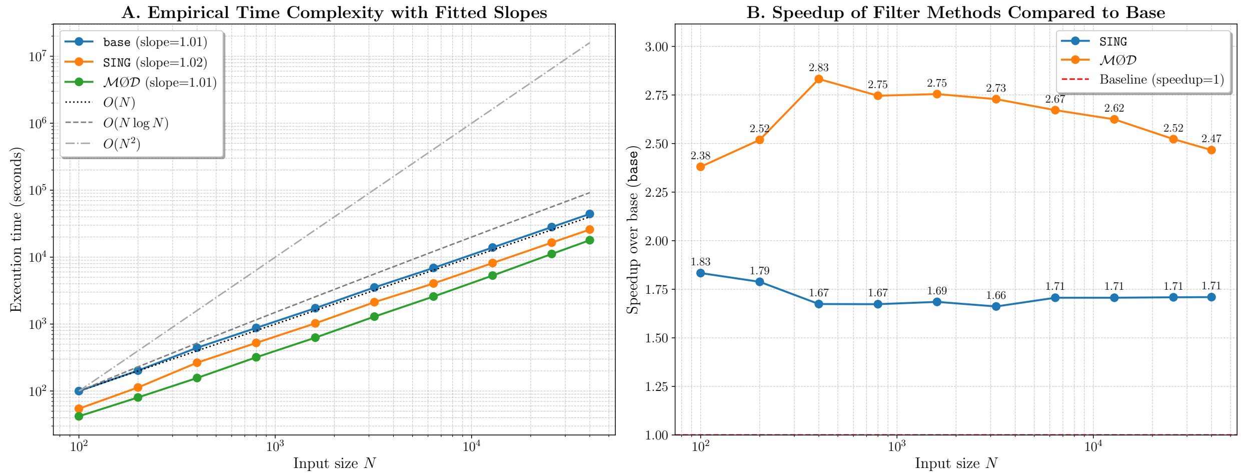

Figure S11: (A) Time-complexity comparison of the *mod* backend, the *nx* backend using its default subgraph search, and SING pre-screening in HTS scenarios. (B) Speedup factors of each filtering method relative to the baseline implementation.

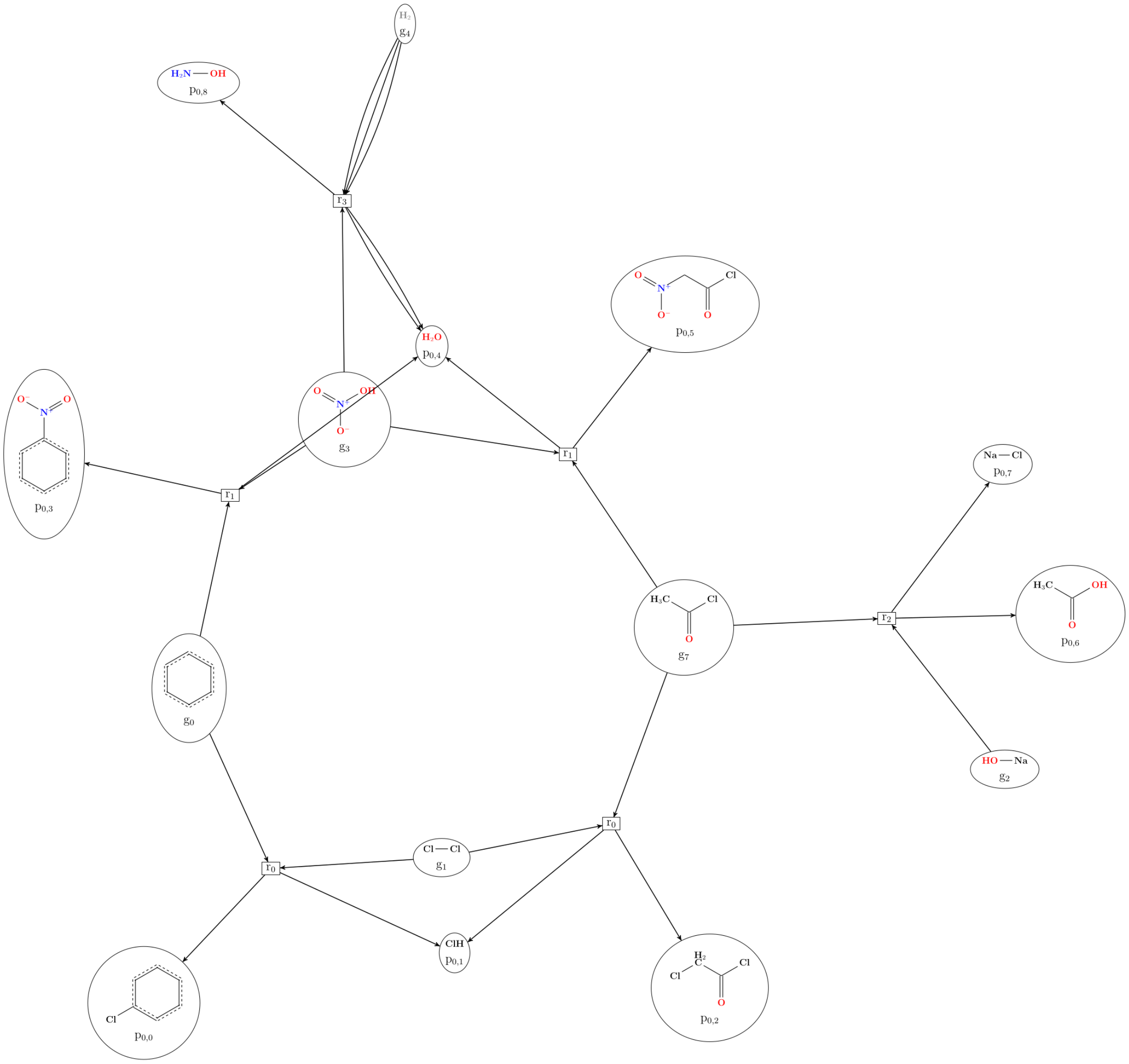

Figure S12: Visualization of a chemical reaction network as a hypergraph, where each molecule is represented by a node and each reaction rule by a hyperedge connecting its reactants and products. The visualization is created using `MØD`.

H Case study

We processed 50,016 reactions from `USPTO_50k` that had been rebalanced with `SynRBL`.[21] Atom maps were generated independently with `RXNMapper`,[22] `GraphormerMapper`[23] and `LocalMapper`.[13] Unlike the default `SynTemp` pipeline,[4] this case study operates on implicit-hydrogen graphs and uses the full corpus rather than splitting into train/validation subsets to exercise end-to-end extraction and reconstruction.

All mapper outputs were standardized and canonicalized with the `Chem` module using implicit-hydrogen representations. Any mapping that produced an empty or invalid AAM was removed. To accelerate preprocessing we used a lightweight Weisfeiler–Lehman hashing option (`WLGH_4` suggested from Section E.3.1) where appropriate and documented the standardization pipeline to ensure determinism.

Ensemble AAM extraction followed a two-stage workflow. First, canonical-match filtering retained 42,384 canonicalized AAMs. Second, nonmatching candidates were revalidated with `Chem.AAMValidator`, recovering additional correct mappings and increasing the ensemble to 44,832 AAMs. Exact-graph deduplication then produced 44,469 unique reactions with fully annotated atom-atom mappings.

Validated AAMs were converted to *Imaginary Transition States* and explicit reaction-center objects with the high-level function `rsmi_to_its` from `IO` module. The same converter supports `GML` export for downstream tools such as `MØD`, but for this study the ITS and reaction-center representations were used directly for clustering and rule extraction.

Reaction centers were clustered with a hybrid WL-hash plus isomorphism strategy using `Graph` module. `WLHash` produced initial buckets and `GraphCluster` performed isomorphism-aware clustering within buckets. The combined hashing and isomorphism pass completed in approximately 13 s for about 45,000 centers on our test hardware. From each isomorphism cluster we selected a representative and constructed `SynRule` objects, yielding a final rule library of **336** rules.

Rules were applied forward and backward with `SynReactor` from `synkit.Synthesis.Reactor`

using implicit hydrogens to avoid mismatches from hydrogen enumeration. Rule application achieved *100%* recovery of the original molecule graphs as measured by graph isomorphism after standardization. The minimal usage pattern for `SynReactor` is shown below and the full reproducible pipeline is available as `case_study.ipynb` in the repository.

```
from synkit.Synthesis.Reactor.syn_reactor import SynReactor
# substrate: molecular graph object
# rc: reaction rule constructed from a reaction center (SynRule)
reactor = SynReactor(
    substrate=substrate,
    rc=rc,
    explicit_h=False,
    implicit_temp=True,
    invert=False
)
results = reactor.smarts    # list of reactions with atom maps
```

I Limitations and Future Work

`SynKit` is intentionally topological and mechanism-oriented: it encodes reactions as graph transformations to support canonicalization, template extraction/application, and clustering on top of `RDKit`/`NetworkX`, with optional hooks to `MØD`. In its present form, it does *not* implement condition-aware scoring (no thermodynamic/kinetic estimators), end-to-end route prioritization, or stereochemical outcome prediction (no explicit handling of regio-/chemo-/enantioselectivity beyond atom-mapped topology). Product prediction is limited to rule-driven candidate generation without internal ranking or conflict resolution among competing outcomes; yield prediction is not provided; and reaction classification is supported only when atom–atom mapping exists (graph/topology features), whereas classifying *unmapped*

reactions into curated taxonomies requires learned models not included here. Condition semantics (solvent, temperature, equivalents, catalysts, pressure, time) are not yet part of the typed graph and appear only as unstructured metadata if present; likewise, stereochemical operators (local-frame definitions, CIP priority evaluation, retention/inversion constraints, conformational prerequisites) are not encoded in the rule system. Uncertainty quantification is absent (no confidence, calibration, or provenance-to-uncertainty mapping), and MTG usage is primarily topological, but do not yet propagate stereochemical state, isotope/fragment labels, or condition-dependent policies across transitions.

To close these gaps, we will add modular capabilities and formalize interfaces without breaking backward compatibility: (i) an external-scoring API that allows *drop-in* ML or heuristic scorers for reaction/product ranking with deterministic seeds, schema validation, and human-readable diffs; (ii) a `SynCat` API enabling classification of unmapped reactions; (iii) interfaces for ML-based yield prediction with recipe-level provenance and optional domain-adaptation hooks; (iv) typed/annotated graphs for stereochemistry, adding local frames and CIP-aware operators, explicit retention/inversion constraints for common motifs (e.g., $\mathtt{S_N1}$/$\mathtt{S_N2}$, `E2`/`E1`), configuration transfer rules across subgraph matches, and validation against curated stereosets; (v) lightweight QM-informed energetics via semiempirical or ML surrogates to provide fast barrier/energy estimates suitable for ranking; (vi) condition handling as first-class attributes on MTG edges (solvent, temperature, equivalents, catalysts), including a minimal schema, normalization utilities, and policy functions that allow scorers to condition on θ; (vii) MTG formalization upgrades: state quotienting with scalable canonicalization, label propagation for isotopes/fragments/roles, partial-order reasoning for concurrency, and incremental construction to reduce memory/runtime. These additions remain optional and pluggable by design so that `SynKit` preserves its core strengths of deterministic mechanistic encoding, dataset hygiene, and interoperability, while progressively enabling experimentally actionable pipelines that integrate ranking, classification, yield estimation, stereochemical reasoning, and condition-aware scoring.

References

(1) Landrum, G.; others RDKit: A software suite for cheminformatics, computational chemistry, and predictive modeling. 2013; `https://www.rdkit.org/`.

(2) Hagberg, A.; Swart, P. J.; Schult, D. A. *Exploring network structure, dynamics, and function using NetworkX*; Los Alamos National Laboratory (LANL), 2007.

(3) Andersen, J. L.; Flamm, C.; Merkle, D.; Stadler, P. F. A Software Package for Chemically Inspired Graph Transformation. Graph Transformation: 9th International Conference, ICGT 2016, Held as Part of STAF 2016, Vienna, Austria, July 5–6, 2016, Proceedings. 2016; pp 73–88.

(4) Phan, T.-L.; Weinbauer, K.; González Laffitte, M. E.; Pan, Y.; Merkle, D.; Andersen, J. L.; Fagerberg, R.; Flamm, C.; Stadler, P. F. SynTemp: Efficient Extraction of Graph-Based Reaction Rules from Large-Scale Reaction Databases. *Journal of Chemical Information and Modeling* **2025**, *65*, 2882–2896.

(5) González Laffitte, M. E.; Weinbauer, K.; Phan, T.-L.; Beier, N.; Domschke, N.; Flamm, C.; Gatter, T.; Merkle, D.; Stadler, P. F. Partial Imaginary Transition State (ITS) Graphs: A Formal Framework for Research and Analysis of Atom-to-Atom Maps of Unbalanced Chemical Reactions and Their Completions. *Symmetry* **2024**, 1217.

(6) Fujita, S. Description of organic reactions based on imaginary transition structures. 1. Introduction of new concepts. *Journal of Chemical Information and Computer Sciences* **1986**, *26*, 205–212.

(7) Wilcox, C. S.; Levinson, R. A. In *Artificial Intelligence Applications in Chemistry*; Pierce, T. H., Hohne, B. A., Eds.; ACS Symposium Series; American Chemical Society: Washington, DC, 1986; Vol. 306; pp 209–230.

(8) Harary, F. *Graph Theory*; CRC Press: Boca Raton, FL, USA, 2018; Reprint / eBook; originally published 1969.

(9) Nugmanov, R. I.; Mukhametgaleev, R. N.; Akhmetshin, T.; Gimadiev, T. R.; Afonina, V. A.; Madzhidov, T. I.; Varnek, A. CGRtools: Python library for molecule, reaction, and condensed graph of reaction processing. *Journal of chemical information and modeling* **2019**, *59*, 2516–2521.

(10) Laffitte, M. E. G.; Beier, N.; Domschke, N.; Stadler, P. F. Comparison of Atom Maps. *MATCH Commun. Math. Comput. Chem.* **2023**, *90*, 75–102.

(11) McKay, B. D.; Piperno, A. Practical graph isomorphism, II. *Journal of symbolic computation* **2014**, *60*, 94–112.

(12) Junttila, T.; Kaski, P. Engineering an efficient canonical labeling tool for large and sparse graphs. 2007 Proceedings of the Ninth Workshop on Algorithm Engineering and Experiments (ALENEX). 2007; pp 135–149.

(13) Chen, S.; An, S.; Babazade, R.; Jung, Y. Precise atom-to-atom mapping for organic reactions via human-in-the-loop machine learning. *Nature Communications* **2024**, *15*, 2250.

(14) Andersen, J. L.; Fagerberg, R.; Flamm, C.; Fontana, W.; Kolcak, J.; Laurent, C. V.; Merkle, D.; Nøjgaard, N. Representing catalytic mechanisms with rule composition. *Journal of Chemical Information and Modeling* **2022**, *62*, 5513–5524.

(15) Amigó, E.; Gonzalo, J.; Artiles, J.; Verdejo, F. A comparison of extrinsic clustering evaluation metrics based on formal constraints. *Information retrieval* **2009**, *12*, 461–486.

(16) Hubert, L.; Arabie, P. Comparing partitions. *Journal of classification* **1985**, *2*, 193–218.

(17) Meilă, M. Comparing clusterings—an information based distance. *Journal of multivariate analysis* **2007**, *98*, 873–895.

(18) Coley, C. W.; Green, W. H.; Jensen, K. F. RDChiral: An RDKit wrapper for handling stereochemistry in retrosynthetic template extraction and application. *Journal of chemical information and modeling* **2019**, *59*, 2529–2537.

(19) Di Natale, R.; Ferro, A.; Giugno, R.; Mongiovì, M.; Pulvirenti, A.; Shasha, D. Sing: Subgraph search in non-homogeneous graphs. *BMC bioinformatics* **2010**, *11*, 1–15.

(20) Corradini, A.; Montanari, U.; Rossi, F.; Ehrig, H.; Heckel, R.; Löwe, M. *Handbook Of Graph Grammars And Computing By Graph Transformation: Volume 1: Foundations*; World Scientific, 1997; pp 163–245.

(21) Phan, T.-L.; Weinbauer, K.; Gärtner, T.; Merkle, D.; Andersen, J. L.; Fagerberg, R.; Stadler, P. F. Reaction Rebalancing: A Novel Approach to Curating Reaction Databases. *Journal of Cheminformatics* **2024**, *16*, 82.

(22) Schwaller, P.; Hoover, B.; Reymond, J.-L.; Strobelt, H.; Laino, T. Extraction of organic chemistry grammar from unsupervised learning of chemical reactions. *Science Advances* **2021**, *7*, eabe4166.

(23) Nugmanov, R.; Dyubankova, N.; Gedich, A.; Wegner, J. K. Bidirectional graphormer for reactivity understanding: neural network trained to reaction atom-to-atom mapping task. *Journal of chemical information and modeling* **2022**, *62*, 3307–3315.
